# When Infodemic Meets Epidemic: Systematic Literature Review

**DOI:** 10.2196/55642

**Published:** 2025-02-03

**Authors:** Chaimae Asaad, Imane Khaouja, Mounir Ghogho, Karim Baïna

**Affiliations:** 1 TICLab College of Engineering and Architecture International University of Rabat Salé Morocco; 2 ENSIAS Alqualsadi, Rabat IT Center Mohammed V University Rabat Morocco; 3 University of Leeds Leeds United Kingdom

**Keywords:** epidemics, social media, epidemic surveillance, misinformation, mental health

## Abstract

**Background:**

Epidemics and outbreaks present arduous challenges, requiring both individual and communal efforts. The significant medical, emotional, and financial burden associated with epidemics creates feelings of distrust, fear, and loss of control, making vulnerable populations prone to exploitation and manipulation through misinformation, rumors, and conspiracies. The use of social media sites has increased in the last decade. As a result, significant amounts of public data can be leveraged for biosurveillance. Social media sites can also provide a platform to quickly and efficiently reach a sizable percentage of the population; therefore, they have a potential role in various aspects of epidemic mitigation.

**Objective:**

This systematic literature review aimed to provide a methodical overview of the integration of social media in 3 epidemic-related contexts: epidemic monitoring, misinformation detection, and the relationship with mental health. The aim is to understand how social media has been used efficiently in these contexts, and which gaps need further research efforts.

**Methods:**

Three research questions, related to epidemic monitoring, misinformation, and mental health, were conceptualized for this review. In the first PRISMA (Preferred Reporting Items for Systematic Reviews and Meta-Analyses) stage, 13,522 publications were collected from several digital libraries (PubMed, IEEE Xplore, ScienceDirect, SpringerLink, MDPI, ACM, and ACL) and gray literature sources (arXiv and ProQuest), spanning from 2010 to 2022. A total of 242 (1.79%) papers were selected for inclusion and were synthesized to identify themes, methods, epidemics studied, and social media sites used.

**Results:**

Five main themes were identified in the literature, as follows: epidemic forecasting and surveillance, public opinion understanding, fake news identification and characterization, mental health assessment, and association of social media use with psychological outcomes. Social media data were found to be an efficient tool to gauge public response, monitor discourse, identify misleading and fake news, and estimate the mental health toll of epidemics. Findings uncovered a need for more robust applications of lessons learned from epidemic “postmortem documentation.” A vast gap exists between retrospective analysis of epidemic management and result integration in prospective studies.

**Conclusions:**

Harnessing the full potential of social media in epidemic-related tasks requires streamlining the results of epidemic forecasting, public opinion understanding, and misinformation detection, all while keeping abreast of potential mental health implications. Proactive prevention has thus become vital for epidemic curtailment and containment.

## Introduction

### Background

The global community braved the COVID-19 crisis, with multiple emerging variants, more than 6 million deaths, and 764 million cases being registered [1]. COVID-19 was dubbed “an individual and collective traumatic event,” and has “directly or indirectly affected every individual in the world” [2]. Four years later, the world is still grappling with the emotional and socioeconomic aftermath of this crisis [3].

However, COVID-19 has not been the first crisis of its kind to affect global public health. Multiple epidemics have spanned the last 2 decades, causing varying degrees of instability and disease burden [4]. An epidemic is defined as “the occurrence in a community or region of cases of an illness, specific health-related behavior, or other health-related events clearly in excess of normal expectancy” [5]. When an epidemic “occurs worldwide or over a very wide area, crosses international boundaries, and affects a large number of people,” it qualifies as a pandemic [5].

Epidemics are often linked to major feelings of uncertainty and loss. The 2014 Ebola outbreak caused rampant fear behaviors in West Africa [6]. The SARS outbreak has created a range of psychiatric conditions, including posttraumatic stress disorder, depressive disorders, and other anxiety spectrum disorders, such as panic, agoraphobia, and social phobia [7]. COVID-19 was associated with major stigma and psychological pressure, further aggravating feelings of guilt, shame, regret, sadness, self-pity, anger, internalized emotions, overwhelmed feelings, negative self-talk, unrealistic expectations, and perceived sense of failure [2]. During epidemics and outbreaks, mistrust of governments and health workers, misinformation, rumors, and conspiracies [8] present challenges to containment and can have a negative impact on mitigation efforts [9-11]. The particular vulnerability surrounding epidemics could render social media users highly suggestible and at risk for fake news acceptance and dissemination [12]. The substantial financial and medical burden imposed by outbreaks and epidemics, in addition to the substantial challenges arising in their progression and aftermath, further complicates the mental health toll they take on the affected population and on vulnerable communities [13].

The control strategies put in place in public health crises to contain the spread of infection are highly dependent on the transmission method and rate [14]. For instance, during COVID-19, various containment measures were adopted, including school closures, shut-downs of nonessential businesses, bans on mass gatherings, travel restrictions, border closures, and curfews [14]. These measures, although necessary for mitigation, can worsen emotional states, contribute to the exacerbation of preexisting socioeconomic inequalities in mental health [15], and lead to unhealthy coping mechanisms, such as problematic internet use, social media addiction, and emotional overeating [16-18].

During epidemics, social media platforms fulfill various functions ranging from informational support to emotional and peer support [19]. They are often a solemn companion offering a tool for connection, a space to grieve, and an instrument of outrage [19]. It is not surprising that the use of social media platforms massively increased during the COVID-19 pandemic [20], rendering them almost essential, ubiquitous, and a catalyst for change, for better and for worse [21].

Social media platforms offer significant amounts of data that can be leveraged for biosurveillance and syndromic surveillance of epidemics and outbreaks [22]. Biosurveillance provides early warning and situational awareness of events using diverse data streams [22]. Efforts directed at facilitating both the early detection and forecasting of disease outbreaks have been increasing in the past 2 decades [22]. Through the analysis of a variety of data sources, syndromic surveillance aims to discern individual and population health indicators before confirmed diagnoses are made [23] using trackable or exhibited behavioral patterns, symptoms, signs, or laboratory findings [23].

Understanding how social media shapes our experiences and preparedness during epidemics, and characterizing the roles it can fulfill, could allow for an improved apprehension of how to efficiently harness this resource for prevention efforts or alleviation of burden of disease [24].

Literature reviews have shown interest in understanding the roles social media fulfills during times of crisis, especially in the last decade [12,25-27]. Social media roles related to the facilitation of public health management, prevention of misinformation, and management of public health behavior and response were found to be of utmost priority [24], and social media topics related to surveillance and monitoring of public attitudes and perceptions, as well as mental health, misinformation, and fake news, were found to be the most well-developed research topics [28]. These 3 particular facets of social media’s intersection with epidemics have not been approached in existing reviews; therefore, a gap remains for the research questions (RQs) proposed in this systematic literature review.

### This Review

This review aimed to examine the “epidemic-social media” relationship and delineate its various aspects, as well as identify the methods used in harnessing social media in epidemics, with a particular focus on monitoring and surveillance, misinformation, and mental health. In light of the current state of global public health, it is vital to understand how a tool as influential as social media can shape the population’s response in times of crisis and how it can be leveraged.

This systematic literature review outlines 3 RQs as follows: (1) How is social media harnessed for epidemic monitoring and management? (RQ1); (2) How is social media used for capturing and managing misinformation during epidemics? (RQ2); and (3) How is social media related to mental health during epidemics? (RQ3).

The remainder of this paper is organized as follows. Methods pertaining to the search strategy and extraction process are detailed in the Methods section*.* Results of the systematic review are synthesized in the Results section*.* Discussion of the major issues and practical implications as well as identified directions for future research are presented in the Discussion section*.* Conclusions are summarized in the Conclusion section*.*

## Methods

### Overview

This systematic review builds upon the preferred reporting items outlined in the PRISMA (Preferred Reporting Items for Systematic Reviews and Meta-Analyses) statement [29] (Multimedia Appendix 1).

### Proposed RQs

The RQs proposed in this systematic literature review examine the epidemic-social media relationship from different perspectives. The first RQ aims to identify potential uses of social media in the context of epidemic management and mitigation. The second RQ examines potential methods used in the context of social media misinformation as it relates to epidemics. Furthermore, the third RQ aims to discern potential aspects of the relationship between social media and public mental health during epidemics.

### Search Strategy

A systematic literature search was undertaken at the beginning of June 2021. A collaborative planning and task allocation process was developed and updated at each stage of the study. The systematic search was conducted across multiple digital libraries—PubMed, IEEE Xplore, ACM Digital Library, ScienceDirect, MDPI, ACL, SpringerLink, arXiv, and ProQuest. Gray literature sources (arXiv and ProQuest) were used to complement the search and reduce publication bias as they provide a venue for authors to share studies with null or negative results that might otherwise not be disseminated.

The RQs were used as a guideline to identify search keywords. The search terms used included “social media” and “epidemics,” with variations depending on the RQ’s objectives and the database searched. For RQ1, the search results of the query (“social media” AND “epidemics”) were complemented by the results of the query (“social media” AND “epidemics” AND “monitoring” AND “tracking”). The combination of these 2 queries allowed for result-filtering without overlimiting the output. The query (“social media” AND “epidemics” AND “fake news”) was used for RQ2. A combination of the queries (“social media” AND “epidemics” AND “mental health” AND “support system”) and (“social media” AND epidemic AND “mental health” AND addiction) was used for RQ3.

These queries were adapted to each database based on its settings. All searches used the parameters *full-text* or *all metadata* in the queries. All searches covered the time range 2010 to 2022.

Table 1 details the number of publications (without duplication) retrieved for screening from each database for each RQ.

**Table 1:** Output of search strategy for research questions (RQs) 1, 2, and 3.

Database	RQ1	RQ2	RQ3
IEEE Xplore	259	27	54
ScienceDirect	1180	371	240
SpringerLink	2189	367	2188
ACL	90	20	121
ACM Digital Library	672	923	1795
MDPI	178	113	70
arXiv	1544	5	0
ProQuest	226	26	127
PubMed	54	149	217

### Study Selection and Data Extraction Strategy

At the initial screening stage, 3 authors assessed the titles and abstracts against the inclusion criteria. Publications included after this screening stage were then retrieved in full-text version, and subsequently screened in the eligibility stage. Three of the authors read the full-text articles independently to ascertain their relevance with regard to the search terms and the research aims. All disagreements on the included articles were resolved by consensus.

To organize the screening process, Rayyan [30], a web application facilitating the collaborative review process and screening process for systematic literature reviews, was used by the authors to import all articles initially collected and screen them following a “blind on” setting, where decisions and labels of any collaborator were not visible to others. Publications with inclusion disagreements were then identified after dropping the “blind on” setting and resolved among authors.

The inclusion and exclusion criteria specified the aims of the review and were agreed upon by all authors (Textbox 1). For a publication to be selected, it needed to address the RQs and be published within the time range. The publication was excluded if it was not a journal paper, conference proceedings paper, or peer-reviewed workshop or symposium paper. Long abstracts and posters were excluded. Publications related to the HIV or tuberculosis epidemic were excluded to preserve the homogeneity of the review. Tuberculosis is a bacterial infection with a high burden of disease, especially in developing countries, while HIV is the virus responsible for AIDS [1]. Both tuberculosis and HIV or AIDS are classified as ongoing worldwide public health issues by the World Health Organization (WHO) and the Centers for Disease Control and Prevention [1]. Given the particularities of both tuberculosis and HIV or AIDS and the high volume of literature review publications related to them [31], the authors agreed to consider both beyond the scope of this review.

**Inclusion and exclusion criteria for the study selection process.**

**Inclusion criteria for studies**

- Within the scope of one of the research questions
- Published between 2010 and 2022
- Relates to an epidemic or pandemic within the last 2 decades
- Includes the use of a social media site
- Is a journal, conference, or workshop paper

**Exclusion criteria for studies**

- Tuberculosis, HIV, or, noninfectious diseases
- Online forums or traditional media
- Book, e-book, letter to editor, magazine, abstracts, case reports, comments, reviews, or poster

In the data extraction stage, the final list of papers was analyzed to answer the RQs and extract pertinent information. The final stage of the PRISMA guidelines [29] was considered in this phase. The following data were extracted from selected papers: authors, publication year, epidemic studied, social media site used, theme, identified method, and key findings. All the related data were extracted independently by 2 investigators. When necessary, differences were resolved by discussing, examining, and negotiating with a third investigator.

### Quality Assessment

The quality of the included studies in this review was appraised using a set checklist of quality criteria. Papers that did not fulfill at least 4 out of the 5 quality criteria were excluded. The checklist was defined as follows:

1. Are the study objectives clearly defined?
2. Are the methods clearly defined and applied?
3. Are the methods applied successfully and correctly?
4. Are accuracy values and efficiency and confidence levels reported?
5. Are limitations clearly reported and adequately represented?
6. Do the contributions outweigh the limitations of the study?

The quality criteria were formulated based on our understanding of the current state of research in this field and the research gap this systematic review is attempting to fill. The papers were assessed for their ability to answer the RQs and enrich the literature while fulfilling quality standards.

Bias was evaluated in this systematic literature review from 2 aspects. First, the risk of bias based on inclusion was limited through the use of multiple reviewers. Second, publication bias was limited by including gray literature which reports negative and null results. To enhance the quality of this review, the authors monitored the planned review tasks and ensured continuous progress monitoring. Collaborative worksheets were created to keep track of scheduled tasks and deadlines, and to note pertinent observations. Validation of the extracted data from selected papers was conducted by the authors and peer-reviewing was maintained at every stage of the systematic review process.

## Results

### Characteristics of the Selected Papers

**Figure 1:**
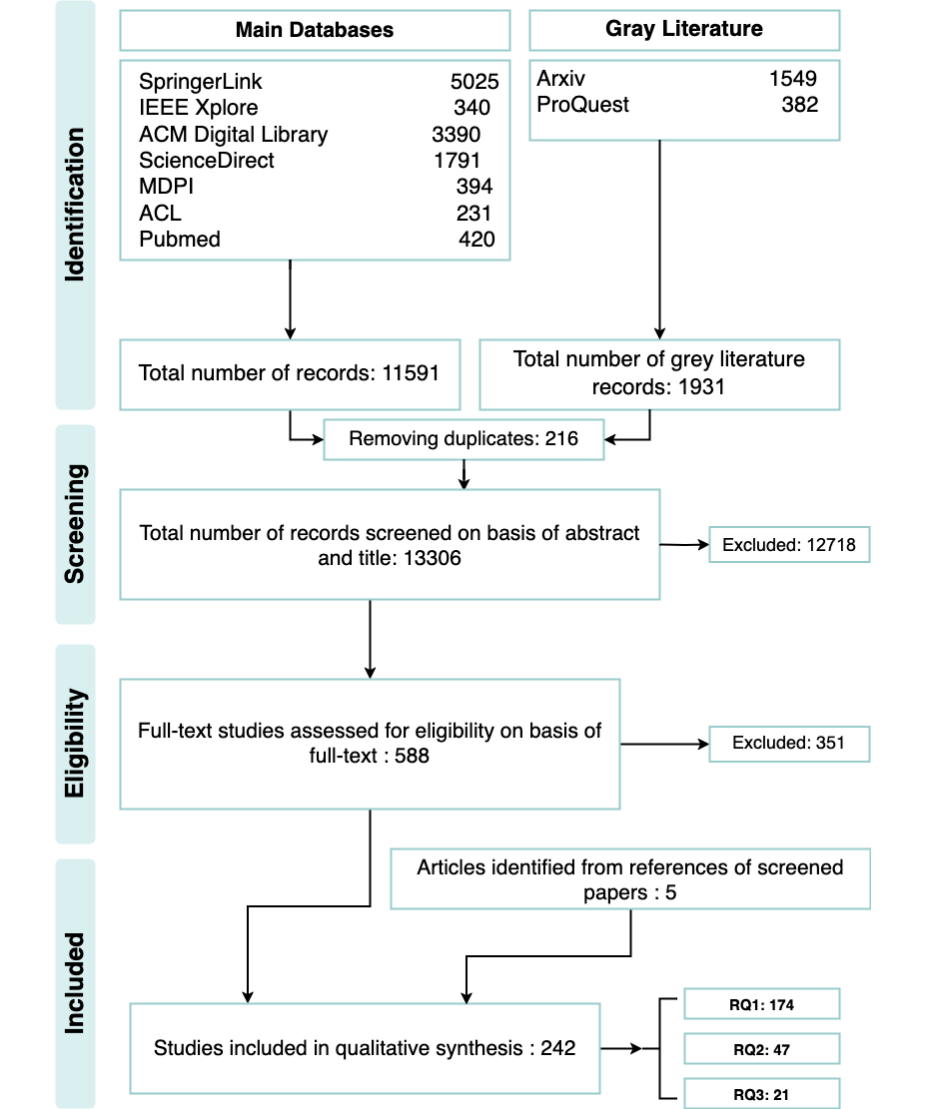
PRISMA (Preferred Reporting Items for Systematic Reviews and Meta-Analyses) flow diagram for the selection of articles of the literature reviewed. RQ: research question.

The search process resulted in a total of 13,522 articles distributed over both the main and gray databases used. After the removal of duplicates, 13,306 (98.4%) titles remained. Of these, 12,718 (95.58%) studies were excluded after the title and abstract screening, as they did not fulfill the inclusion criteria. A flow diagram of the results of literature collection, screening, eligibility, and inclusion is presented in Figure 1.

Of the 588 studies that were full-text screened, 351 (59.7%) did not meet the inclusion criteria. 5 (1.4%) papers were identified from reference lists of included papers. A total of 242 (67.9%) studies were selected for inclusion in this review as summarized in subsection Answers to RQs.

**Figure 2:**
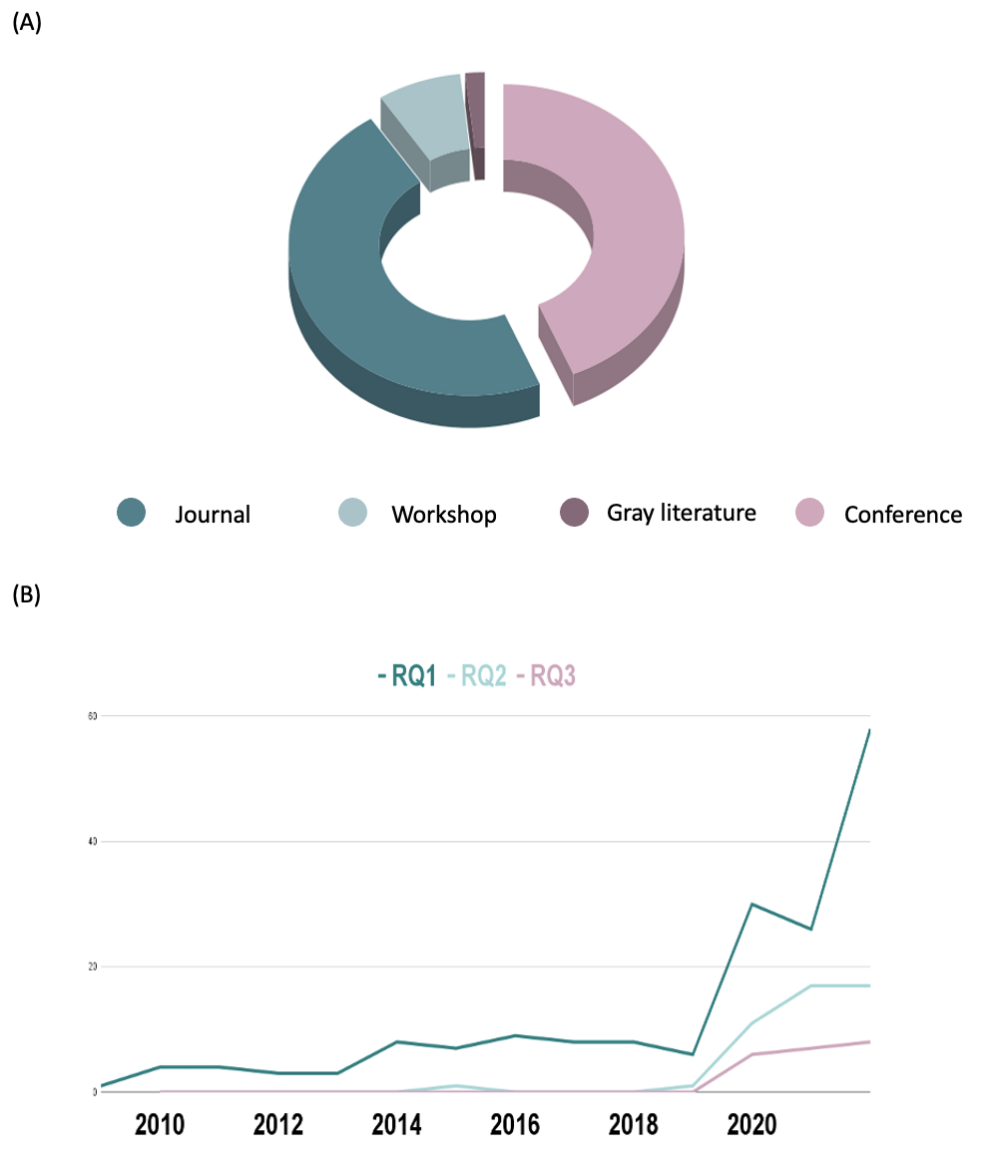
Distribution of selected papers by (A) type and (B) year. RQ: research question.

The papers included in the review were distributed as follows: 47.1% (114/242) were journal papers, 43.8% (106/242) were publications of conference proceedings, 7.4% (18/242) were workshop and symposium publications, while 1.7% (4/242) were gray literature (Figure 2A).

The publications spanned from 2010 to 2022. As can be seen in Figure 2B, the number of publications peaked in 2020 and continued to increase for all RQs. All the selected papers that answeredRQ3 spanned the from 2020 to 2021. A similar distribution was seen in papers that answeredRQ2, where selected papers were from 2015, 2019, 2020, and 2021. RQ1, which studies the aspects of epidemic management and mitigation using social media, included the highest number of papers and spanned the entire decade.

### Social Media Platforms Used

**Figure 3:**
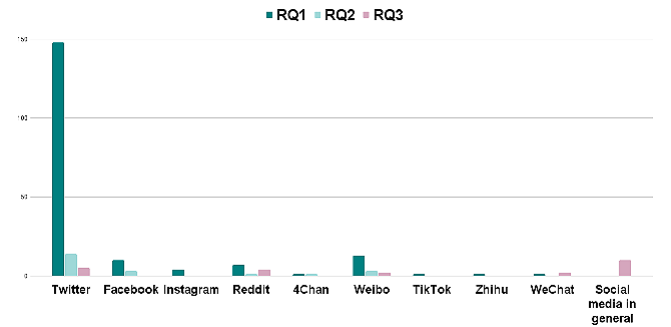
Number of selected publications using each social media platform included in the systematic literature review. RQ: research question.

Several social media platforms were used in the literature selected for this systematic review. X (formerly Twitter) is one of the most widely used platforms for sharing “microblogs.” These short messages are called tweets and can take up to 280 characters. In contrast, Weibo, is a popular platform to share and discuss individual information and life activities as well as celebrity news in China. As can be seen in Figure 3, X followed by Weibo seems to be the platform of choice for most works aiming to study epidemic monitoring and mitigation through social media (RQ1) and epidemic-related misinformation on social media (RQ2). For epidemic and social media–related mental health aspects, most works seem to take a generalist approach rather than a platform-specific one. Compared with other social media sites, such as Facebook and Instagram, which predominantly include heterogeneous posts, X offers a more concise “microblog” format.

### Epidemics Studied

**Figure 4:**
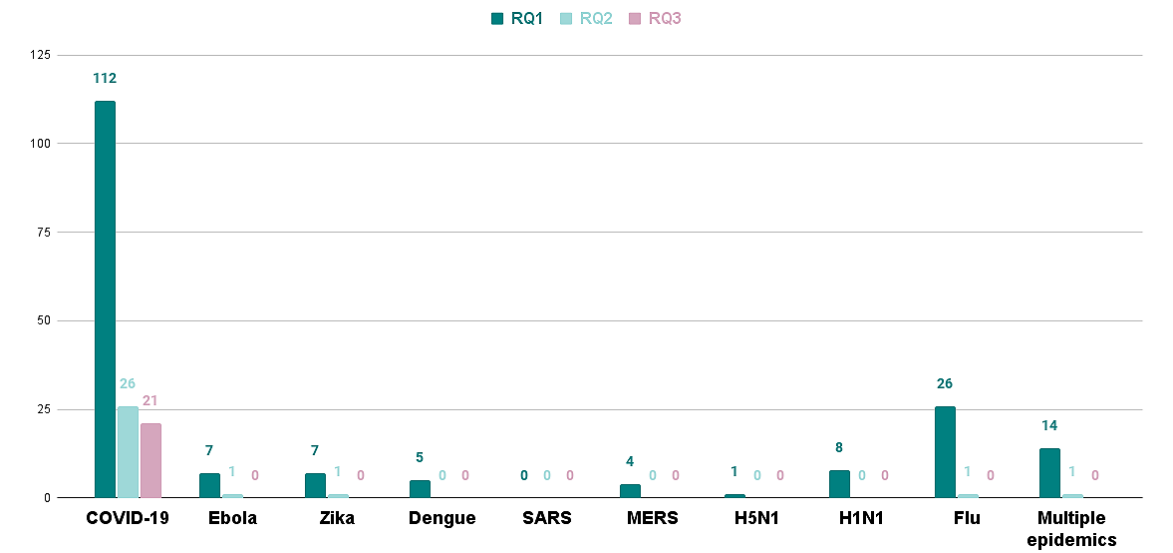
Number of selected publications pertaining to each epidemic included in the systematic literature review. MERS: Middle East respiratory virus; RQ: research question.

**Figure 5:**
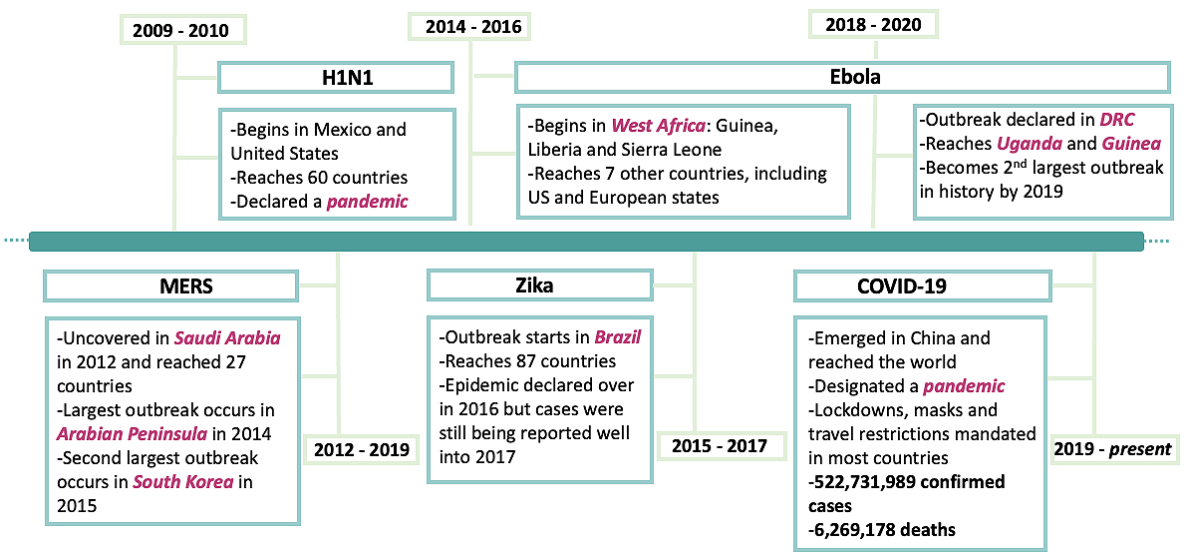
Timeline of the epidemics and pandemics spanning the last decade and included in the systematic literature review. SARS is pre-2009 and dengue fever has caused multiple outbreaks. Both are not illustrated in the timeline but are included in the systematic literature review. DRC: Democratic Republic of the Congo; MERS: Middle East respiratory syndrome.

The selected literature discussed multiple epidemics (Figures 4 and 5), including vital hemorrhagic fevers and influenza-like illness (ILI).

Dengue fever and Zika fever are mosquito-borne diseases caused by the dengue virus and Zika virus, respectively, and spread by several species of female mosquitoes of the Aedes genus [1]. The disease is now endemic in more than 100 countries with potential risk in other areas [1,32]. The WHO declared the Zika outbreak of 2016 and the Ebola outbreak in 2019 as public health emergencies of international concern (PHEICs) [1].

ILI is a nonspecific respiratory illness characterized by fever, fatigue, cough, and other symptoms. Cases of ILI can be caused either by influenza strains or by other viruses, such as coronaviruses. Influenza remains a global and year-round disease burden and causes illnesses that range in severity and sometimes lead to hospitalization and death. Seasonal influenza epidemics are mainly caused by influenza A and B viruses [1]. The influenza A virus subtype strain H1N1, commonly referred to as the swine flu, disproportionately affects children and younger people. H1N1 was declared a PHEIC in 2009 and then designated a pandemic [1]. Coronaviruses include SARS, MERS (Middle East respiratory syndrome), which can be contracted through direct or indirect contact with infected animals [1], as well as COVID-19 caused by the SARS-CoV-2 virus. The latter was designated a PHEIC and a pandemic by the WHO. As of April 26, 2023, the official death toll from COVID-19 reached 6,915,268 [1].

The highest number of selected publications for all RQs related to COVID-19, followed by influenza (Figure 4). This trend is due, in part, to the volume of the COVID-19 research output [33].

### Answers to RQs

#### Overview

A thematic analysis of the selected literature was conducted with the aim of identifying the main themes of each RQ. Themes were identified following the objectives of the paper and its results. For each theme, papers were organized by method, social media platform used, and the epidemic studied. Methods were grouped categorically. For instance, content analysis includes automated, linguistic, thematic, qualitative, or quantitative analysis, while dictionary-based classification entails a lexicon-based classification. Machine learning (ML) classification includes conventional ML models, while deep learning (DL) entails methods based on artificial neural networks with representation learning. Although it must be acknowledged that overlaps exist, the categorization used in this paper is based on the most distinctive and predominant use or theoretical approach of each method. This categorization is meant to facilitate a structured analysis and discussion of the literature by grouping papers according to their primary methodological approach, thus allowing a clearer comparison and contrast of their contributions, strengths, and limitations.

#### RQ1. Social Media for Epidemic Monitoring and Management

##### Overview

Social media platforms offer significant amounts of data, which can be potentially useful in biosurveillance and syndromic surveillance of epidemics and outbreaks.

Two main themes were identified in the selected papers that addressed how social media could be used in epidemic management, namely, (1) *epidemic surveillance and forecasting*, and (2) *public opinion understanding*.

##### Epidemic Surveillance and Forecasting

Several works proposed a dictionary-based classification of X for the surveillance of COVID-19 [34,35], dengue fever [36,37], Ebola [38,39], H1N1 [40,41], influenza [42-50], Zika [51,52], MERS [53], and a combination of epidemics [53-55]. Similar epidemic surveillance applications using dictionary-based classification were conducted using data from Weibo for Ebola [56] and influenza [57,58], Reddit for Zika [59], and Facebook for MERS and other epidemics [53].

Reported results indicated that epidemic surveillance can be achieved using varying strategies. For instance, social distancing–related tweets can be grouped into categories, such as implementation, purpose, social disruption, and adaptation, and used to quantify the spatiotemporal prevalence and evolution of COVID-19 social distancing on X [34]. Similarly, official social media channels of information and health organizations, such as the Centers for Disease Control and Prevention, WHO, and National Institutes of Health (NIH) can be monitored, and their X data can be classified to recognize “alarming” news and “concerning” news [35]. Dengue fever reported surveillance strategies to include systems aggregating social media data with weather and flood information [36], and using volume, location, time, and public perception as spatiotemporal dimensions [37].

Results also reported keyword-based data extraction and classification as a strategy for the creation of an Ebola monitoring platforming in China using Weibo data [56] and in Africa using X data [39], and for multiple epidemics [53-55].

Regression analysis was reported to be used for tracking and forecasting influenza [46,47,49,57] and Zika [52], and the Markov switching model was used for real-time early-stage influenza detection with emotion factors for epidemic and nonepidemic segmentation [58]. Statistical analysis was used to study the relationship between human activities collected from Sina Weibo and morbidity patterns and at-risk areas during COVID-19 in China [60].

Correlation analysis reported that X, in addition to other sources, could not provide an Ebola alert more than a week before the WHO and that X’s message volume was correlated more with news article volume than with the number of Ebola cases [38].

Additional dictionary-based surveillance methods include quantitative analysis, filtering, and normalization of X data for H1N1 [40,41] and Zika [51]; mathematical modeling of influenza trends using geo-tagged X streams [42]; time series for X symptom reporting matching ILI [43]; keyword analysis for Zika risk assessment [59,61] as well as influenza risk surveillance [44] and condition aggravation [45], and upcoming influenza spike detection [50], sentiment analysis [48].

Different methodologies using conventional ML were reported to be used for dengue-related event monitoring [62] and lazy associative classification [63]; influenza detection [64-66]; influenza activity monitoring [67]; seasonal influenza trend prediction [68,69]; ILI prevalence prediction [70] and awareness or infection classification [71]; location-specific influenza state detection [58,72,73]; avian influenza outbreak detection [74]; disease-related category classification [75-77] for Ebola, MERS, and dengue fever; guideline-related category classification for ILIs [78]; supervised text classification [79]; topic classification for symptomatic manifestation and prevention of mosquito-borne diseases [80]; infectious disease analytics [81,82] and COVID-19 case forecasting [83], and X-enabled contact tracing [84] and early detection [85].

Conventional ML models used for epidemic surveillance and monitoring include support vector machine (SVM), naive Bayes (NB), and logistic regression (LR).

Several DL techniques were applied for epidemic monitoring [86-90]; fine-tuning of semisupervised model with unlabeled COVID-19 dataset [91]; disease-infected individual detection in tweets using bidirectional encoder representations from transformers (BERT)–based model and disease-infection region identification using spatial analysis [92]; classification of Zika- and Ebola-related tweets [93], COVID-19 related tweets [94], and influenza-related information [95,96]; H1N1 outbreak forecasting and individual-level disease progressing using semisupervised multilayer perceptron and a online stochastic training algorithm [97]; and correlation of X reports on H1N1 infectious disease control using gray wolf optimizer and least square method [98]. Results reported that mathematical modeling can be used to understand the influence of X on the spread of H1N1 [98,99] and information entropy to quantify the impact of social network information [100].

Results showed that social network theory and social network analysis can be used for the prediction of infected groups and early detection of contagious outbreaks in social media [101-104], and that topic modeling techniques, such as latent Dirichlet allocation (LDA) can be used for epidemic intelligence [105-110] to detect major epidemic-related events [111], monitor information spread [112], and rank epidemic-related tweets [113].

##### Understanding Public Opinion

Several methods were used in the selected literature to extract and analyze public opinions expressed on social media. These methods were based on content analysis of social media data, linguistic analysis, qualitative analysis, lexicon-based analysis, sentiment analysis, valence aware dictionary and sentiment reasoner–based sentiment analysis, topic modeling, conventional ML models, and DL models.

Social media content analysis was used to analyze public discourse around H1N1- [114] and Zika-related risks [61], inspect social media coverage related to influenza vaccinations [115] and COVID-19 vaccinations [116-127], measure public health concerns [128], identify stances toward policies, such as social distancing and face masks [129], identify emotional composition of online discourse before and after COVID-19 [130], and inspect the presence and escalation of negative sentiments toward China [131]. Latent semantic analysis and LDA were used to mine opinions on X related to the hashtag #IndiaFightsCorona [132]. Topic detection and sentiment analysis were performed for opinion mining, concern exploration, and public opinion analysis in the context of epidemics [133-142], and for pattern analysis [143-145]. Social media content analysis was also used for tracking information spread [146], narratives and information voids [147], monitoring engagement [148-152] and emotional response [153-161], requests for medical assistance [162], health behavior changes [163], governmental response [164,165], and physicians’ opinions [166].

Public reaction tracking and investigation were performed using SVM and NB for topic and sentiment analysis [127,167-175]; SVM, NB, and random forest (RF) for social media content classification (eg, caution, advice, notifications, donations, etc) [176]; crisis analysis [177,178]; clustering for topic extraction [179]; and LR for prevention category tweet classification [180]. ML was used to analyze public discourse against masks [181], extract insights on policy response [182], and understand expressions of help-seeking during COVID-19 [183].

BERT-based models were used for public sentiment assessment of data related to COVID-19 available in X [184-186]. Multilingual COVID-19 emotion prediction was performed using a fine-tuned BERT “BERTmoticon” [187], while bias and user opinion were identified using a GPT [188]. A language model for Arabic Moroccan dialect was used for topic modeling, emotion recognition, and polarity analysis [189]. long short-term memory (LSTM), BERT, and enhanced language representation with informative entities were used to analyze the evolution of sentiments in the face of the public health crisis due to COVID-19 [190]. Bi-LSTM with an attention mechanism was used for sentiment analysis of COVID-19–related tweets [191]. Term-frequency analysis was adopted to build an emerging topic graph [192], while the k-means algorithm, LR, SVM, and NB were used to identify COVID-19–related topics [193]. An extra tree and convolutional neural network-based ensemble model was reported to have outperformed conventional ML models in a sentiment classification task [194]. French COVID-19 tweet classification was performed using FlauBERT [195], while opinion monitoring was achieved using a combination of LSTM and global vectors for word representations [196]. Convolutional neural network was used for COVID-19 personal health mention detection [197].

Findings of analyses performed in the context of Ebola, Zika, and influenza revealed that social media posts from health organizations were highly effective when incorporating visuals and that public response was more affected by these communications when they acknowledged the concerns and fear of the community [198]. In the context of Ebola, findings highlighted that online blame was directed toward the affected populations as well as figures with whom social media users had preexisting political frustrations [199].

Analysis of X discussions in relation to COVID-19 revealed the presence of negative sentiments and an association between the words “coronavirus” and “China” [200]; a gradual increase in calls for social distancing, quarantining, and working from home among social media users [201]; a growing number of anger expressions directed at individuals refusing sanitary protocols; and the frequent use of the words “family,” “life,” “health,” and “death” [201]. Analysis of X hashtags also revealed categories, such as quarantine, panic buying, school closures, lockdowns, frustration, and hope [201], as well as mentions of mental health issues and gratitude for essential workers [201]. Other categories and themes identified or used for manual annotation of topics discussed on social media include resource provision, employment and strategies [87], statistics, prevention, hygiene, diagnosis, politics, world news [202], conspiracy, economy, mortality, origin, and outbreak [203].

Findings also indicated increased levels of connectivity and agency coordination during the early-stage response to COVID-19 [87]. Disregarding COVID-19–imposed sanitary and government recommendations was potentially linked to uncertainty in times of crisis, overwhelm by “noise” presented on social media, and varying socioeconomic factors [204].

Results revealed that social media analytics were an efficient approach to capture the attitudes and perceptions of the public during COVID-19 as mentioned in studies by Yigitcanlar et al [205] and Xia et al [206]. Fear and collectivism were identified as predictors of people’s preventive intention in the context of COVID-19 [207]. “Sadness” appeared to spike after the WHO declared COVID-19 as a pandemic, while “anger” and “disgust” spiked after the death toll surpassed the hundred thousand in the United States [187].

Tables 2 and 3 summarize the methods, epidemics, and social media used in studies pertaining to epidemic forecasting and prediction and understanding of public opinion.

**Table 2:** Summary of methodologies used in studies addressing the first part of research question 1 (epidemic surveillance and forecasting).

Method, epidemic studied, and social media used				References
**Dictionary-based classification**				
	**COVID-19**			
		X (formerly Twitter)	[34,35]	
		Sina Weibo	[60]	
	**Dengue fever**			
		X	[36,37]	
	**Ebola**			
		X	[38,39]	
		Weibo	[56]	
	**H1N1 or swine flu**			
		X	[40,41]	
	**Influenza or flu**			
		X	[42-50]	
		Sina Weibo, Tancent Weibo	[57,58]	
	**Zika**			
		X	[51,52]	
		Reddit	[59]	
	**MERS^a^**			
		X	[53]	
		Facebook	[53]	
	**Multiple epidemics**			
		X	[53-55]	
		Facebook	[53]	
**ML^b^ classification**				
	**Dengue fever**			
		X	[62,63]	
	**Influenza or flu**			
		X	[64-73]	
		Sina Weibo, Tancent Weibo	[58,73]	
		Facebook	[68]	
	**H1N1 or swine flu**			
		X	[78]	
	**H5N1 or avian influenza**			
		X	[74]	
	**MERS**			
		X	[75-77]	
	**Ebola**			
		X	[93]	
	**Zika**			
		X	[93]	
	**COVID-19**			
		X	[83-85]	
	**Multiple epidemics**			
		X	[75-77,79-82]	
**DL^c^ classification**				
	**COVID-19**			
		X	[35,86,88-91,94]	
	**Ebola**			
		X	[93]	
	**Zika**			
		X	[93]	
	**Influenza or flu**			
		X	[95]	
	**Multiple epidemics**			
		X	[92]	
**Mathematical modeling**				
	**COVID-19**			
		WeChat	[100]	
	**H1N1 or swine flu**			
		X	[97-99]	
**Social network analysis**				
	**COVID-19**			
		X	[101,102]	
	**Influenza or flu**			
		Facebook	[104]	
	**Multiple epidemics**			
		X	[103]	
**Topic modeling**				
	**COVID-19**			
		X	[88,102,105,106]	
	**Dengue fever**			
		X	[111]	
	**Ebola**			
		X	[112]	
	**Influenza or flu**			
		X	[107]	
		Weibo	[108]	
	**Zika**			
		X	[109]	
	**Multiple epidemics**			
		X	[113]	

^a^MERS: Middle East respiratory syndrome.

^b^ML: machine learning.

^c^DL: deep learning.

**Table 3:** Summary of methodologies used in studies addressing the second part of research question 1 (understanding public opinion).

Method, epidemic studied and social media used				References
**Content analysis**				
	**COVID-19**			
		X	[87,123,126,141,144,146,148,151,156,157,159,160,200,201]	
		Instagram	[202]	
		Reddit	[155,157,204]	
		TikTok	[122]	
		Weibo	[151,162]	
		Facebook	[126,149]	
	**Ebola**			
		X	[198,199]	
		Facebook	[199]	
		Instagram	[198]	
	**Zika**			
		Reddit	[61]	
	**Influenza or flu**			
		X	[115]	
		Facebook	[115]	
	**H1N1 or swine flu**			
		X	[115]	
		Facebook	[115]	
**Dictionary-based classification**				
	**COVID-19**			
		X	[128,129,147,153,158,165,166,203,205,206]	
		Facebook	[147]	
		Instagram	[147]	
		Reddit	[147]	
		Weibo	[129]	
	**H1N1 or swine flu**			
		X	[114]	
**ML^a^ classification**				
	**COVID-19**			
		X	[127,132,164-167,177-179,181]	
		Weibo	[176]	
		Facebook	[182]	
		Instagram	[182]	
	**Zika**			
		X	[180]	
**DL^b^ classification**				
	**COVID-19**			
		X	[118-120,130,184-189,191,194-197]	
		Weibo	[190]	
**Topic modeling**				
	**COVID-19**			
		X	[116,133-135,138,143,154,163,189,192,193]	
		Reddit	[139]	
		Weibo	[136,137]	
		Zhihu	[183]	
**Social network analysis**				
	**COVID-19**			
		X	[117,121,124,125,140,142,145,150,152]	
		Facebook	[161]	

^a^ML: machine learning.

^b^DL: deep learning.

#### RQ2. Social Media for Misinformation Management During Epidemics

##### Overview

Misinformation, or “fake news,” has become a social phenomenon and has received increased attention in the past few years. Although the term, “fake news” has been around since the 1890s [208], the emergence and exponential rise in popularity of social media platforms has brought the term to the “front page.” Fake news can fall into multiple categories depending on the intent and form it takes [208]. For instance, fake news can be false information and rumor fabrication (eg, celebrity gossip), hoaxes (eg, doomsday 2012), conspiracy theories (Q-Anon), and satire (eg, The Onion). The intent can range from deception for the purposes of monetary or personal gain to satirizing real news.

One main theme was identified in the selected papers that addressed how social media could be used in misinformation management during epidemics, namely, misinformation detection and characterization. Three subsequent subthemes were identified based on the scope of selected literature, namely: fake news identification, fake news characterization, and information distortion and conspiracy theories.

##### Misinformation Detection and Characterization

###### Overview

The selected literature focused on the inspection of news or claims shared on social media, with the aim of classifying them based on trustworthiness. Several methods were used to analyze social media content and detect misleading information, such as expert annotation, DL models, and social network analysis. While some papers focused on technical approaches to the detection of fake news, other studies tried to identify various characteristics related to the source or propagation of fake news.

###### Fake News Identification

Several works performed fake news identification using DL models [209-211] with conventional ML models for comparison or as baselines. A modified 3-layer-each LSTM and gated recurrent unit were used along with 6 conventional ML classification models (decision trees, LR, k-nearest neighbors, RF, SVM, and NB) on a “Covid-19 fake news Twitter dataset” [212] to identify fake news [210]. Findings reported that the best test results were obtained by LSTM (2 layers), with an accuracy of 98.6%, a precision of 98.55%, a recall of 98.6%, and an *F*_1_-score of 98.5% [210]. Similarly, a multilayer perceptron, LR, decision trees, RF, NB, SVM, and gradient boosting were used for COVID-19 fake news detection in tweets and concluded that RF outperformed other models with an accuracy of 78%, a recall of 100%, a precision of 85%, and an *F*_1_-score of 83% [211]. Expert annotated tweets were used to evaluate the performance of a BERT-based misinformation detection system [213]. Findings suggest that knowledge about the domain vocabulary helps domain-adapted models in predicting the correct stance, as it did for retrieval.

Detecting misleading and fake news was also performed by several studies using methods based on pretrained transformer models, bi-LSTM networks, artificial neural networks, convolutional neural networks, deep transfer learning [214-220], and using hybrid methodologies [221-227].

A semisupervised probabilistic graphical model that aimed to jointly learn the interactions between user trustworthiness, content reliability, and post credibility for influenza posts’ credibility analysis outperformed baseline models (RF and Bayesian network) with an accuracy of 71.7% on data from Sina Weibo [209]. LR was performed on a small dataset of Facebook comments to detect fake news [228]. Several ML models, including gradient boosting classifier, LR, RF classifier, and decision tree classification were used in multiple works for fake news classification on social media [229-233].

Other works seeking to curtail the misinformation of COVID-19–related news and support reliable information dissemination used manual analysis through fact-checkers as well as consensus to verify the veracity and correctness of selected tweets and social media posts [234,235]. This is illustrated in a use case analyzing Facebook and X content in both English and Amharic [234] and an Ebola study [235].

###### Fake News Characterization

A manual annotation of tweet sources following 5 categories (academic, government, media, health professional, and public) allowed for the creation of a gold standard dataset for training a LR model based on 6 million Arabic tweets related to infectious viruses, such as MERS and COVID-19 [236]. Rumor detection using a top-down strategy consisting of extracting posts associated with previously identified rumors reported an 84.03% accuracy for the LR classifier [236]. Higher precision was obtained at the expense of higher runtime using ML models [232]. Similarly, topic modeling based on the k-means algorithm was used to identify sources of COVID-19–related rumors [193]. An entropy-based method was used to investigate the potential control of COVID-19 rumors [237] and content analysis was used to evaluate rumor dissemination and official responses during COVID-19 [238].

Semantic correlations between textual content and attached images were mined using a pretrained convolutional neural network to learn image representations and use them to enhance textual representations and train a fake news detector [239].

Content analysis showed that fake news from multiple sources could be classified using a taxonomy of health and non–health-related types and reported that the response of the public health system was debilitated by the propagation of fake news [240]. Roots of misinformation were categorized as politically related, false medical information, celebrity and pop culture related, religious belief related, and fraud and criminality related [241]. A comparison of fake news sources between China, Iran, and the United States showed that fake science is the main “root” of misinformation in China, while counterexpertise, that is, the rejection of mainstream academic expertise, politically motivated and governmentally sourced misinformation is the most prevalent source of fake news in the United States. In Iran, discourse about COVID-19 was found to be politically manipulated by the government, while official religious figures hindered the dissemination of accurate information [241]. Statistical analysis found bias of sentiment in fake news, as well as biases of gender of the user and media use with respect to real news [242].

Bot detection using BERT was performed as a potential strategy to improve fake news detection [243]. Findings imply that the ratio of real news to fake news is very similar between human accounts and bot accounts, and bot detection could not improve the performance of the fake news detection model [243].

Findings of an information mutation study using A Lite BERT reported that misinformation propagation could potentially be exacerbated by user commentary and found a positive association between information mutation and spreading outcome [244].

The findings of a propagation analysis showed that false claims propagate faster than partially false claims and that tweets containing misinformation are more often concerned with discrediting other information on social media [245].

An investigation leveraging neural networks and quantitative content analysis that aimed to reveal the conditions that lead audiences to accept and disseminate a fake claim as it relates to the Zika virus showed that Zika tweets, including threat cues and protection cues, are positively associated with the likelihood of sharing fake news [246]. In addition, findings of a descriptive analysis showed that the quality of news sources varies considerably with regard to information on COVID-19 [247], Results of a computational analysis indicated that the COVID-19 infodemic is highly characteristic of community structure, shaped by ideological orientation, typology of fake news, and geographic areas of reference [248]. Data from X indicated that content could be labeled according to political affiliation, media source, and type of source (political, satire, mainstream media, science, conspiracy or junk science, clickbait, and fake or hoax) [248].

###### Information Distortion and Conspiracy Theories

Information distortion in X cascades was found to be linked to oversimplification, distortion of logical links, omission of facts, and a shift in the medical topic to political and business disputes [249]. Risk amplification by information dramatization appeared to be linked to controversial topics as well as social and cultural influences [250].

Manual content and semantic analysis and topic modeling (LDA) techniques of tweet content were conducted through an examination of key term distribution, context, and medical terminology verification [249]. In a COVID-19 5G conspiracy use case, LDA and social network analysis were used to identify several topics from dataset of tweets [251] related to “5G conspiracy” and “5G threat” and discuss topics, including 5G towers, radiation effects, network, and radiation [252,253]. Emerging COVID-19–related conspiracy theories were detected by estimating narrative networks with an underlying graphical model and using a collection of data from Reddit subreddits and 4Chan threads related to the pandemic [254]. Findings identified multiple central conspiracy theories illustrated by examples, such as incorporating the COVID-19 conspiracy into Q-Anon conspiracy, #scamdemic and #plandemic [255], 5G as the cause of COVID-19 [252,253], antivax conspiracy, Bill Gates, #filmyourhospital conspiracy [256], and Pizzagate conspiracy [254]. Table 4 summarizes the methods, epidemics, and social media used in studies pertaining to misinformation management and detection.

**Table 4:** Summary of methods used in papers addressing research question 2–misinformation identification and characterization.

Method, epidemic studied, and social media used			References
**ML^a^ classification**			
	**COVID-19**		
		X	[193,210,211,221-227,229-232]
		Facebook	[228,230]
		Sina Weibo	[233]
	**Multiple epidemics**		
		X	[236]
**DL^b^ classification**			
	**COVID-19**		
		X	[210,213-218,220-227,239]
		Facebook	[156]
		Instagram	[217]
		Weibo	[219,239]
**Topic modeling**			
	**COVID-19**		
		X	[237,249,252,255]
**Social network analysis**			
	**COVID-19**		
		X	[248,252,253,256]
		Reddit, 4Chan	[254]
**Probabilistic graph modeling**			
	**Influenza**		
		Weibo	[209]
**Manual content analysis**			
	**COVID-19**		
		X	[234,241,248,249]
		Facebook	[234,241,247]
		Weibo	[241,250]
		Instagram	[241]
	**Ebola**		
		X	[235]
**Quantitative content analysis**			
	**COVID-19**		
		X	[213,242,245]
		Weibo	[250]
	**Zika**		
		X	[246]

^a^ML: machine learning.

^b^DL: deep learning.

#### RQ3. Social Media’s Relationship With Mental Health During Epidemics

##### Overview

During the implementation of restrictive measures requiring limited social contact, social media can become one of the few methods to safely engage with others, rendering it the sole support system of vulnerable populations. Mental health deterioration can manifest in expressions shared on the internet and be used to gauge the toll epidemics and subsequent containment strategies could potentially take on individuals.

Two main themes were identified in the selected papers addressing how social media can be integrated in aspects of public mental health management during epidemics, namely, (1) social media as a tool to gauge the mental health toll of epidemics, and (2) impact of social media consumption during epidemics on mental health.

##### Mental Health Assessment Using Social Media

Assessment of mental health state was performed using conventional ML [257-259], DL [260-262], and topic modeling techniques [263,264]. Psychological profiles of Weibo users were predicted using ML and online ecological recognition with emotional measures and cognitive indicators, such as anxiety, depression, Oxford happiness, social risk judgment, and life satisfaction [257]. LSTM was used to estimate the rate of depression in the population during the COVID-19 pandemic using Reddit data [260]. Topic modeling, expert intervention, and X data were used to evaluate the possible effects of critical factors related to COVID-19 on the mental well-being of the population in a psychological vulnerability study [263].

Findings revealed that negative emotional indicators of psychological traits increased in anxiety and depression after COVID-19 was declared an epidemic or pandemic [257,262], while life satisfaction and happiness decreased [257]. A 53% average increase in depression rate of Reddit users was noted in selected months after the pandemic [260], and negative psychological vulnerability manifested in negative emotions toward social distancing and hospitalization [263]. Financial burden was found to increase the odds of depressive nonsuicidal thoughts for individuals who suffered job loss during COVID-19 [264]. Results indicated the beginning of recovery following the immediate mental health impact of the COVID-19 pandemic [259].

Table 5 summarizes the methods, epidemics, and social media used in studies pertaining to the use of social media as a tool to gauge the mental health toll of epidemics.

**Table 5:** Summary of methods used in papers addressing the first part of research question 3 (mental health assessment using social media).

Method and epidemic studied		Social media used	References
**ML^a^ classification**			
	**COVID-19**		
		Weibo	[257]
		X	[258,259]
		Reddit	[259]
**DL^b^ classification**			
	**COVID-19**		
		Reddit	[260,261]
		X	[262]
**Topic modeling**			
	**COVID-19**		
		X	[263,264]
		Reddit	[264]

^a^ML: machine learning.

^b^DL: deep learning.

##### Association of Social Media Consumption and Mental Health

Multiple papers conducted cross-sectional studies and statistical analysis to investigate the association between social media consumption and mental health complications during epidemics, particularly during COVID-19. Several studies relied on regression analysis, online surveys, the Generalized Anxiety Disorder Scale, and the Patient Health Questionnaire.

Findings revealed that frequent Sina Weibo use was associated with higher anxiety, depression, and a combination of both [265], and compulsive WeChat use was associated with social media fatigue, emotional stress, and social anxiety [266]. Frequent use of WeChat during COVID-19 was also associated with depression and secondary trauma and was found to be a significant predictor of both [19], while close contact with individuals with COVID-19, along with spending ≥2 hours daily on COVID-19–related news on WeChat was associated with probable anxiety and depression in community-based adults [267]. The association between social media consumption and anxiety and depression was found to be statistically significant [265,268,269] and positively associated with emotional overeating and anxiety in individuals with neuroticism [18].

The association between the mental health of students receiving higher education and social media use during COVID-19 confinement was analyzed, and results indicated that students in the 18 to 24 years age group, who were not in a relationship and who had lower academic results, presented the highest levels of addiction to social media [16]. Significant positive associations were found between relatedness, need, frustration, and social media addiction, as well as between social media addiction, depressive symptoms, and loneliness [17]. Excessive social media use was also found to fully mediate the relationship between COVID-19–related life concerns and schizotypal traits [270].

Appropriate guidance of adolescents in the use of social networking sites was found to have a potential impact on the mitigation of negative emotions during the COVID-19 pandemic [271].

On the positive side, social media use was found to be rewarding for Wuhan’s residents through information sharing and emotional and peer support [19]. Social media breaks were reported to have the potential to promote well-being during the COVID-19 pandemic [19]. In addition, positive mental health and mindfulness appeared to serve as protective factors, and positive mental health was found to be a mediator between the COVID-19 burden and addictive social media use [272].

Table 6 summarizes the methods, epidemics, and social media used in studies pertaining to the association of social media use with mental health issues during epidemics.

**Table 6:** Summary of methods used in papers addressing the second part of research question 3 (association of social media consumption with mental health).

Method, epidemic studied, and social media used				References
**Statistical analysis**				
	**COVID-19**			
		WeChat	[19,266]	
		Sina Weibo	[265]	
		Social media in general	[16-18,267-273]	

## Discussion

### Principal Findings

This systematic literature review conceptualized 3 RQs to investigate if, when, and how social media can be harnessed for successful epidemic management and mitigation, effective curtailment of fake news propagation, and a refined understanding of social media’s relationship with mental health during epidemics. It presented a systematic categorization and summary of methods, social media sites, and epidemics broached in the 242 selected works and identified potential research directions and practical implications related to the RQs.

Papers selected pertaining to RQ1 comprised the highest number of papers and included publications from all years of the decade, illustrating continuous and ongoing efforts by the scientific community to harness social media’s potential for improved containment measures during epidemics.

COVID-19 was found to be the epidemic most studied in selected papers. This is due to the rapid increase of COVID-19–related publications since the first year of the pandemic. The frequency of publication and the volume of the academic output contributed to the creation of the COVID-19 Open Research Dataset [33]. A similar rising trend was seen in RQ2. This can be explained by the emergence of the “fake news” phenomena on social media and its particular increase in times of crisis. The selected publications answering RQ3 were published from 2020 to 2022. Papers that pertained to RQ3 were much lesser in number than those that pertained to RQ1 and RQ2. Given the mental health aspect of this particular RQ, a potential inference can be made suggesting a very recent interest in mental health as it relates to social media and epidemics. X was found to be the most used social media site in the selected literature, potentially suggesting its attractiveness to works conducting linguistic analysis and classification tasks. This can also be due to the differences in the popularity of social media sites by geographic location and key demographics. The availability of application programming interfaces to crawl data is also a major factor in choosing specific social media platforms as data sources.

### General Discussion

The systematic literature review presented in this paper differs from existing reviews and aims to cover a different gap in the literature. Existing works have taken an interest in a broader range of crises, including noninfectious diseases and health risk behaviors [12], disasters in general [25], and new and reemerging infectious diseases [26]. Focus was directed toward effectively targeting vulnerable populations to test interventions and improve health outcomes [12], collective behavior [25], and generalized perspectives on emergency situations [27]. Differences other than scope include data sources, time range, and volume of literature. The review presented in this paper covered a broader time range, included gray literature, and reviewed a sizable volume of research papers.

The review’s findings indicated that social media was found to be an effective way to understand the public’s reactions and engagement during epidemics [205]. Monitoring topics of discussion during epidemics allowed for insights on whether aspects of epidemic management needed improvement, whether the public agrees with government decisions, and which emotions are linked to the onset of epidemics and mitigation protocols [198,204-206]. Analysis of opinions related to aspects, such as COVID-19 vaccinations were proposed and could be used to give feedback to governments and health organizations to implement better suited protocols [116,122,124-126] for mitigation, and to identify topics of misinformation, and therefore offer clarifications or conduct further awareness efforts to combat rumors and conspiracies [254]. Results also indicated that social media can be used in case forecasting [83], X-enabled contact tracing [84], early detection [85], tracking adherence to preventive guidelines, such as wearing masks and social distancing [205,206], and monitoring symptomatic self-expressions of infection [80]. Misinformation detection on social media was performed as a classification task, manually using experts and fact checkers, and using artificial intelligence techniques; however, presented several challenges. Misinformation often used language styles of academics and health professionals to deceive the public [236] and propagated faster when it included higher levels of threat due to the collective stress reaction it generated [246]. “Troll” accounts were found to play the second most prominent role is misinformation spread and present a “substantial cause for concern” [248]. Other challenges of misinformation detection related to limitations of studies due to the use of small batches of data [252], false positives [228], and a “politicization” of neutral health emergency crises [235].

Although epidemics were found to cause negative emotions and mental health issues [260,262,263], many expressions of positive emotions were noted [257], reflecting group cohesiveness rather than pure personal emotions. Group threats contributed to the manifestation of more beneficial behaviors and social solidarity [269]. Viewing heroic acts, speeches from experts, and knowledge of the disease and prevention methods were associated with more positive effects and less expressions of depression [269]. Media content, including useful information for self-protection was found to be potentially helpful to people during epidemics and may enhance active coping, prevention behaviors, and instill a sense of control [269]. The use of social media during epidemics, although linked with manifestations of anxiety and depression, appeared to benefit Wuhan residents and was perceived as an important activity during lockdown [19]. Balancing social media use to obtain ample informational as well as emotional and peer support, while avoiding the potential mental health toll, is a difficult task for users, especially without the availability of alternative and easily accessible sources of health information [19].

Using social media data for mental health assessment has its challenges and limitations. It can add a population or demographic bias to results, given that some social media sites are predominantly used by younger people or are more or less popular depending on the country [257,263]. Depending on the social media site (eg, Reddit), the user pool skews younger, and thus could be more prone to depression [260]. Moreover, some analyses are based on a weekly basis, with a relatively large granularity, which has certain influences on reflecting the changing trend of social mentality in a timely manner [257]. The qualitative nature of the results obtained and interpreted by domain experts limits the generalization of the findings and requires more corroborating results. Consequently, findings may need additional data to be strengthened [260,263]. As for works pertaining to the association of social media consumption with psychological outcomes, a causal link has not been established due to the cross-sectional nature of the contributions. Studies reflected a single point in time for participants, therefore, further longitudinal studies are necessary. In addition, the surveys were conducted on the web, and consequently, respondent bias is possible [265]. The recruitment of all participants from the same country and from one social media platform can introduce bias to studies [266,268], in addition to potential gender biases and sample representativeness [18,19], and recall bias related to self-reporting [269]. The results could not exclude the possibility of residual confounding caused by unmeasured factors.

A change can be seen in the evolution of research themes over time and through different epidemics. A sizable number of works focused on the influenza epidemic surveillance using lexicon-based and dictionary-based classifications, as well as classical ML techniques. This volume of literature could potentially be linked to the influenza prediction “wave” that preceded, paralleled, and followed the dereliction of the “Google flu trend” after its failure to predict major outbreaks [274]. Although various methods were used, ML and DL techniques were most frequently used for COVID-19 surveillance. Scientific contributions evolved with the emergence of more epidemics. COVID-19 appeared to have benefited from the digitization of literature as well as the development and improvements taking place in the fields of natural language processing, ML, DL, social network analysis, and topic modeling. The global nature of the COVID-19 crisis generated an influx of publications and contributions. The theme of misinformation management has also evolved with epidemics and with the proliferation of social media fake news, bots, troll accounts, and widely propagated conspiracy theories. COVID-19 has been the subject of multiple controversies and conspiracies, which encouraged scientific efforts to study potential curtailment methods. As for the mental health aspect, all publications pertaining to the scope of RQ3 were related to COVID-19, and it appeared that previous epidemics were not subject to social media association analysis. This could be due to the fear linked to COVID-19 and the challenging nature of sanitary measures such as global lockdowns and social distancing, which led to an increase in social media reliance. It could also be due to the decade’s zeitgeist which brought online mental health discussions and awareness front and center.

### Identified Issues

One of the major issues identified was the lack of preemptive measures building on the results of previous studies and aiming to implement social media–enabled processes in real time or near real time. Lessons learned are not efficiently integrated in crisis mitigation measures nor used as building blocks for optimized proactive prevention. A synergy between government health agencies, research communities, and the public would allow for the success of social-media public health initiatives. Such collaborative efforts require effective and trustworthy interactions. This highlights an additional issue related to the relative inefficiency of social media campaigns. Populations need to be targeted for both informative purposes and for active emotional support. Understanding public opinion is useful to gauge sentiments and reactions, and therefore it is important to remedy the gap for applications integrating extracted opinions in targeted epidemic management.

Because of the medical and financial burden of epidemics, mental health concerns are often ignored by both governments and the public. As a result, the manifestation of several mental health–related symptoms becomes more prevalent as epidemics progress. In the case of the Ebola outbreak in 2014, symptoms of posttraumatic stress disorder and anxiety-depression were more prevalent even after a year of the Ebola response [199]. When limited resources are geared for epidemic containment, the health care system focuses majorly on emergency services. Therefore, individuals with substance abuse and dependency disorders may see deterioration in their mental health [13]. During community crises, event-related information is often sought in an effort to retain a sense of control in the face of fear and uncertainty and their psychological manifestations. When misleading misinformation is propagated on social media, perceptions of risk are distorted, leading to extreme public panic, stigmatization, and marginalization [13]. Psychological interventions and psychosocial support would have a direct impact on the improvement of public mental health during epidemics.

### Directions for Future Research

We identified several issues and gaps in the literature related to the RQs of this systematic literature review and suggest potential paths for future research.

Given the recognized impact of epidemics on mental health and the prevalent use of social media platforms during times of crisis, it is necessary to explore the aspects of social media leading to mental health deterioration during epidemics. Potential factors range from increased consumption levels of social media, social media addiction, emotional fatigue due to overwhelm, and consumption of “sad” content. Investigating which aspects of social media use are responsible for worsening states of mental health and mental health disorders would allow a targeted approach to curbing this negative impact during times of crisis. As for health-related fake news, it is important to understand what makes citizens prone to engaging in fake news sharing. Specifically, features identifying both an individual’s and a group’s susceptibility to believe and share misinformation need to be determined and categorized. Levels of education, geographic and demographic profiles, cultural influences, and psychological vulnerability are potential features requiring further investigation in their association with fake news dissemination on social media and within communities.

Epidemics are rapidly changing phenomena requiring fast interventions and decision-making. Although postcrisis analysis is imperative for an improved understanding of lessons learned, proactive epidemic management is vital and would have the most impact on mitigation efforts. Integrating artificial intelligence techniques into this proactive surveillance could further optimize this process.

In addition, misinformation propagation has a significant impact on the success of interventions given that both the components of exaggerated fear and apathy linked to misinformation can hinder management efforts. However, the investigation of misinformation needs to be extended to include potential links between misinformation and mental health deterioration.

### Practical Implications

This work has several potential practical implications pertaining to different entities.

*Implications for governing entities* include the development of an efficient misinformation correction strategy to fight incorrect information, rumors, and conspiracy theories related to epidemics; the development of clear communication channels for knowledge dissemination to build trust with the public; the development of interventions to limit the impact of epidemics on stress responses (anxiety, depression) due to distorted risk perceptions; the bolstering of public awareness efforts on sanitary measures and proactive protection; and the insurance of the supply of medical staff available to treat patients, as well as psychological support staff to assist patients and their families in navigating the ramifications of infection and loss of loved ones.

*Implications for social media platforms* include taking a leadership position in the management of epidemic-related fake news by implementing built-in fact-checking processes and assisting health agencies and scientific entities in disseminating factual information about the disease, its symptoms, its potential risk, and efficient sanitary measures for the public to adopt.

*Implications for the public* include improving community resilience during epidemics using social media groups and assisting in combating misinformation.

### Limitations

The results of this review should be considered in light of several limitations. The data sources used in this review did not cover all existing scientific databases, and therefore, cannot generalize findings to the entirety of the literature. The scope of the review focused on specific aspects of the epidemic-social media relationship, and so does not provide a general overview. Although the process of data extraction and analysis was undertaken with extreme diligence, there can be potential for bias. Despite our recognition of the inherent limitations of any search strategy, we have ensured our commitment to the rigor and transparency of the systematic review process.

### Conclusions

Given the collective experience of epidemics, responses by communities can often provide insight into the degree of adherence toward preventive measures as well as mitigation protocols. In an effort to control the spread of epidemics, governments, public health institutions, and health care professionals generally issue guidelines for the public through online portals, news sources, and in the past decade, social media. Online “chatter” can indicate the public’s response to these guidelines, and their sentiments toward the epidemic itself or specific topics related to it, such as vaccinations, treatments, mortality rates, etc. Mitigation efforts require collaborative strategies and public involvement; therefore, gaining insight into public opinion and response can prove vital in the success or failure of such efforts.

It is evident that epidemic preparedness and mitigation protocols need to be adjusted to deal with the special challenges that accompany the technological revolution taking place, especially in light of the considerable impact of the ongoing infodemic. In addition, it is vital to have effective ways to exploit the full potential of social media without risking the toll it could potentially take on users’ mental health. The systematic literature review presented in this paper covers several key aspects of the relationship between epidemics and social media, especially with respect to fake news and mental health. Methods used to answer RQs are categorized. The findings of this review could shed light on broader implications related to data quality concerns and privacy considerations in epidemic surveillance, thus highlighting the lack of works proposing ethical, legal, and technical frameworks to accompany scientific efforts. Learning from past crises and integrating a digital and social media-enabled infrastructure into public health protocols could make a difference in future preparedness levels.

## Data Availability

The datasets generated during and analyzed during this study are available in the Github repository [275].

## Appendix

Multimedia Appendix 1 PRISMA (Preferred Reporting Items for Systematic Reviews and Meta-Analyses) checklist.

## Abbreviations

BERT: bidirectional encoder representations from transformers
DL: deep learning
ILI: influenza-like illness
LDA: latent Dirichlet allocation
LR: logistic regression
LSTM: long short-term memory
ML: machine learning
NB: naive Bayes
PHEIC: public health emergency of international concern
PRISMA: Preferred Reporting Items for Systematic Reviews and Meta-Analyses
RF: random forest
RQ: research question
SVM: support vector machine
WHO: World Health Organization

## References

[1] Health topics. World Health Organization.

[2] Mukhtar S (2020). Psychological health during the coronavirus disease 2019 pandemic outbreak. Int J Soc Psychiatry.

[3] Duarte Alonso A, Kok SK, Bressan A, O'Shea M, Sakellarios N, Koresis A, Buitrago Solis MA, Santoni LJ (2020). COVID-19, aftermath, impacts, and hospitality firms: an international perspective. Int J Hosp Manag.

[4] Major epidemics of the modern era. Council on Foreign Relations.

[5] Porta M (2008). A Dictionary of Epidemiology.

[6] Shultz JM, Baingana F, Neria Y (2015). The 2014 Ebola outbreak and mental health: current status and recommended response. JAMA.

[7] Mak IW, Chu CM, Pan PC, Yiu MG, Chan VL (2009). Long-term psychiatric morbidities among SARS survivors. Gen Hosp Psychiatry.

[8] Saroj A, Pal S (2020). Use of social media in crisis management: a survey. Int J Disaster Risk Reduct.

[9] Lin TH, Chang MC, Chang CC, Chou YH (2022). Government-sponsored disinformation and the severity of respiratory infection epidemics including COVID-19: a global analysis, 2001-2020. Soc Sci Med.

[10] Pavela Banai I, Banai B, Mikloušić I (2022). Beliefs in COVID-19 conspiracy theories, compliance with the preventive measures, and trust in government medical officials. Curr Psychol.

[11] Pfeffer B, Goreis A, Reichmann A, Bauda I, Klinger D, Bock MM, Plener PL, Kothgassner OD (2022). Coping styles mediating the relationship between perceived chronic stress and conspiracy beliefs about COVID-19. Curr Psychol.

[12] Charles-Smith LE, Reynolds TL, Cameron MA, Conway M, Lau EH, Olsen JM, Pavlin JA, Shigematsu M, Streichert LC, Suda KJ, Corley CD (2015). Using social media for actionable disease surveillance and outbreak management: a systematic literature review. PLoS One.

[13] Roy A, Singh AK, Mishra S, Chinnadurai A, Mitra A, Bakshi O (2021). Mental health implications of COVID-19 pandemic and its response in India. Int J Soc Psychiatry.

[14] Kaimann D, Tanneberg I (2021). What containment strategy leads us through the pandemic crisis? An empirical analysis of the measures against the COVID-19 pandemic. PLoS One.

[15] Serrano-Alarcón M, Kentikelenis A, Mckee M, Stuckler D (2022). Impact of COVID-19 lockdowns on mental health: evidence from a quasi-natural experiment in England and Scotland. Health Econ.

[16] Oliveira AP, Nobre JR, Luis H, Luis LS, Pinho LG, Albacar-Riobóo N, Sequeira C (2022). Social media use and its association with mental health and internet addiction among Portuguese higher education students during COVID-19 confinement. Int J Environ Res Public Health.

[17] Cheng C, Lau YC (2022). Social media addiction during COVID-19-mandated physical distancing: relatedness needs as motives. Int J Environ Res Public Health.

[18] Gao Y, Ao H, Hu X, Wang X, Huang D, Huang W, Han Y, Zhou C, He L, Lei X, Gao X (2022). Social media exposure during COVID-19 lockdowns could lead to emotional overeating via anxiety: the moderating role of neuroticism. Appl Psychol Health Well Being.

[19] Zhong B, Huang Y, Liu Q (2021). Mental health toll from the coronavirus: social media usage reveals Wuhan residents' depression and secondary trauma in the COVID-19 outbreak. Comput Human Behav.

[20] (2021). Social media and COVID-19: a global study of digital crisis interaction among Gen Z and Millennials. World Health Organization.

[21] Kapoor KK, Tamilmani K, Rana NP, Patil P, Dwivedi YK, Nerur S (2017). Advances in social media research: past, present and future. Inf Syst Front.

[22] Walters RA, Harlan PA, Nelson NP, Hartley DM, Voeller JG (2010). Data sources for biosurveillance. Wiley Handbook of Science and Technology for Homeland Security.

[23] Mandl KD, Overhage JM, Wagner MM, Lober WB, Sebastiani P, Mostashari F, Pavlin JA, Gesteland PH, Treadwell T, Koski E, Hutwagner L, Buckeridge DL, Aller RD, Grannis S (2004). Implementing syndromic surveillance: a practical guide informed by the early experience. J Am Med Inform Assoc.

[24] Selerio E Jr, Caladcad JA, Catamco MR, Capinpin EM, Ocampo L (2022). Emergency preparedness during the COVID-19 pandemic: modelling the roles of social media with fuzzy DEMATEL and analytic network process. Socioecon Plann Sci.

[25] Eismann K, Posegga O, Fischbach K (2016). Collective behaviour, social media, and disasters: a systematic literature review. Proceedings of the European Conference on Information Systems.

[26] Tang L, Bie B, Park SE, Zhi D (2018). Social media and outbreaks of emerging infectious diseases: a systematic review of literature. Am J Infect Control.

[27] Abdulhamid NG, Ayoung DA, Kashefi A, Sigweni B (2020). A survey of social media use in emergency situations: a literature review. Inf Dev.

[28] Michailidis PD (2022). Visualizing social media research in the age of COVID-19. Information.

[29] Page MJ, McKenzie JE, Bossuyt PM, Boutron I, Hoffmann TC, Mulrow CD, Shamseer L, Tetzlaff JM, Akl EA, Brennan SE, Chou R, Glanville J, Grimshaw JM, Hróbjartsson A, Lalu MM, Li T, Loder EW, Mayo-Wilson E, McDonald S, McGuinness LA, Stewart LA, Thomas J, Tricco AC, Welch VA, Whiting P, Moher D (2022). [The PRISMA 2020 statement: an updated guideline for reporting systematic reviewsDeclaración PRISMA 2020: una guía actualizada para la publicación de revisiones sistemáticas]. Rev Panam Salud Publica.

[30] Ouzzani M, Hammady H, Fedorowicz Z, Elmagarmid A (2016). Rayyan-a web and mobile app for systematic reviews. Syst Rev.

[31] Taggart T, Grewe ME, Conserve DF, Gliwa C, Roman Isler M (2015). Social media and HIV: a systematic review of uses of social media in HIV communication. J Med Internet Res.

[32] Guzman MG, Harris E (2015). Dengue. The Lancet.

[33] Wang LL, Lo K, Chandrasekhar Y, Reas R, Yang J, Eide D, Funk K, Katsis Y, Kinney R, Li Y, Liu Z, Merrill W, Mooney P, Murdick D, Rishi D, Sheehan J, Shen Z, Stilson B, Wade A, Wang K, Wang NX, Wilhelm C, Xie B, Raymond D, Weld DS, Etzioni O, Kohlmeier S CORD-19: the COVID-19 open research dataset. arXiv.

[34] Kwon J, Grady C, Feliciano JT, Fodeh SJ (2020). Defining facets of social distancing during the COVID-19 pandemic: Twitter analysis. J Biomed Inform.

[35] Rao HR, Vemprala N, Akello P, Valecha R (2020). Retweets of officials' alarming vs reassuring messages during the COVID-19 pandemic: implications for crisis management. Int J Inf Manage.

[36] Othman MK, Danuri MS (2016). Proposed conceptual framework of Dengue Active Surveillance System (DASS) in Malaysia. Proceedings of the International Conference on Information and Communication Technology.

[37] Gomide J, Veloso A, Meira W Jr, Almeida V, Benevenuto F, Ferraz F, Teixeira M (2011). Dengue surveillance based on a computational model of spatio-temporal locality of Twitter. Proceedings of the 3rd International Web Science Conference.

[38] Yom-Tov E (2015). Ebola data from the internet: an opportunity for syndromic surveillance or a news event?. Proceedings of the 5th International Conference on Digital Health 2015.

[39] Marc T, Kodzo A (2020). How useful are social networks for analyzing epidemics? The example of Twitter for monitoring the 2018–2019 Ebola epidemic in Africa. Proceedings of the International Multi-Conference on: “Organization of Knowledge and Advanced Technologies”.

[40] Szomszor M, Kostkova P, de Quincey E (2010). #Swineflu: Twitter predicts Swine Flu outbreak in 2009. Proceedings of the Third International Conference on Electronic Healthcare.

[41] Quincey D, Kostkova P (2009). Early warning and outbreak detection using social networking websites: the potential of Twitter. Proceedings of the Second International ICST Conference on Electronic Healthcare.

[42] Chon J, Raymond R, Wang H, Wang F (2015). Modeling flu trends with real-time geo-tagged Twitter data streams. Proceedings of the 10th International Conference on Wireless Algorithms, Systems, and Applications.

[43] Stilo G, Velardi P, Tozzi AE, Gesualdo F (2014). Predicting flu epidemics using Twitter and historical data. Proceedings of the International Conference on Brain Informatics and Health.

[44] Hwang MH, Wang S, Cao G, Padmanabhan A, Zhang Z (2013). Spatiotemporal transformation of social media geostreams: a case study of Twitter for flu risk analysis. Proceedings of the 4th ACM SIGSPATIAL International Workshop on GeoStreaming.

[45] Talvis K, Chorianopoulos K, Kermanidis KL (2014). Real-time monitoring of flu epidemics through linguistic and statistical analysis of Twitter messages. Proceedings of the 9th International Workshop on Semantic and Social Media Adaptation and Personalization.

[46] Lampos V, Zou B, Cox IJ (2017). Enhancing feature selection using word embeddings: the case of flu surveillance. Proceedings of the 26th International Conference on World Wide Web.

[47] Lampos V, Bie TD, Cristianini N (2010). Flu detector-tracking epidemics on Twitter. Proceedings of the Machine Learning and Knowledge Discovery in Databases.

[48] Byrd K, Mansurov A, Baysal O (2016). Mining Twitter data for influenza detection and surveillance. Proceedings of the International Workshop on Software Engineering in Healthcare Systems.

[49] Comito C, Forestiero A, Pizzuti C (2018). Improving influenza forecasting with web-based social data. Proceedings of the IEEE/ACM International Conference on Advances in Social Networks Analysis and Mining.

[50] Kostkova P, Szomszor M, St. Louis C (2014). #swineflu: the use of Twitter as an early warning and risk communication tool in the 2009 swine flu pandemic. ACM Trans Manage Inf Syst.

[51] Abouzahra M, Tan J (2021). Twitter vs. Zika—the role of social media in epidemic outbreaks surveillance. Health Policy Technol.

[52] Masri S, Jia J, Li C, Zhou G, Lee MC, Yan G, Wu J (2019). Use of Twitter data to improve Zika virus surveillance in the United States during the 2016 epidemic. BMC Public Health.

[53] Chaudhary S, Naaz S (2017). Use of big data in computational epidemiology for public health surveillance. Proceedings of the International Conference on Computing and Communication Technologies for Smart Nation.

[54] Romano S, Di Martino S, Kanhabua N, Mazzeo A, Nejdl W (2016). Challenges in detecting epidemic outbreaks from social networks. Proceedings of the 30th International Conference on Advanced Information Networking and Applications Workshops.

[55] Ji X, Chun SA, Geller J (2012). Epidemic outbreak and spread detection system based on Twitter data. Proceedings of the First International Conference on Health Information Science.

[56] Feng S, Hossain L (2016). Weibo surveillance of public awareness to Ebola disaster in China. Proceedings of SAI Intelligent Systems Conference (IntelliSys) 2016.

[57] Zhang Q, Gioannini C, Paolotti D, Perra N, Perrotta D, Quaggiotto M, Tizzoni M, Vespignani A (2015). Social data mining and seasonal influenza forecasts: the FluOutlook platform. Proceedings of the European Conference on Machine Learning and Knowledge Discovery in Databases.

[58] Sun X, Ye J, Ren F (2014). Real time early-stage influenza detection with emotion factors from Sina Microblog. Proceedings of the Fifth Workshop on South and Southeast Asian Natural Language Processing.

[59] Gui X, Kou Y, Pine KH, Chen Y (2017). Managing uncertainty: using social media for risk assessment during a public health crisis. Proceedings of the 2017 CHI Conference on Human Factors in Computing Systems.

[60] Yuan M, Liu T, Yang C (2022). Exploring the relationship among human activities, COVID-19 morbidity, and at-risk areas using location-based social media data: knowledge about the early pandemic stage in Wuhan. Int J Environ Res Public Health.

[61] Gui X, Kou Y, Pine K, Ladaw E, Kim H, Suzuki-Gill E, Chen Y (2018). Multidimensional risk communication: public discourse on risks during an emerging epidemic. Proceedings of the 2018 CHI Conference on Human Factors in Computing Systems.

[62] Souza RC, de Brito DE, Cardoso RL, de Oliveira DM, Meira W Jr, Pappa GL (2014). An evolutionary methodology for handling data scarcity and noise in monitoring real events from social media data. Proceedings of the 14th Ibero-American Conference on AI.

[63] Souza RC, Assunção RM, de Oliveira DM, de Brito DE, Meira W Jr (2016). Infection hot spot mining from social media trajectories. Proceedings of the European Conference on Machine Learning and Knowledge Discovery in Databases.

[64] Zhang F, Luo J, Li C, Wang X, Zhao Z (2014). Detecting and analyzing influenza epidemics with social media in China. Proceedings of the 18th Pacific-Asia Conference on Advances in Knowledge Discovery and Data Mining.

[65] Culotta A (2010). Towards detecting influenza epidemics by analyzing Twitter messages. Proceedings of the First Workshop on Social Media Analytics.

[66] Wakamiya S, Kawai Y, Aramaki E (2016). After the boom no one tweets: microblog-based influenza detection incorporating indirect information. Proceedings of the Sixth International Conference on Emerging Databases: Technologies, Applications, and Theory.

[67] Hassan Zadeh A, Zolbanin HM, Sharda R, Delen D (2019). Social media for nowcasting flu activity: spatio-temporal big data analysis. Inf Syst Front.

[68] Achrekar H, Gandhe A, Lazarus R, Yu SH, Liu B (2012). Online social networks flu trend tracker: a novel sensory approach to predict flu trends. Proceedings of the 5th International Joint Conference on Biomedical Engineering Systems and Technologies.

[69] Aramaki E, Maskawa S, Morita M (2011). Twitter catches the flu: detecting influenza epidemics using Twitter. Proceedings of the Conference on Empirical Methods in Natural Language Processing.

[70] Zhang K, Arablouei R, Jurdak R (2017). Predicting prevalence of influenza-like illness from geo-tagged tweets. Proceedings of the 26th International Conference on World Wide Web Companion.

[71] Lamb A, Paul MJ, Dredze M (2013). Separating fact from fear: tracking flu infections on Twitter. Proceedings of the 2013 Conference of the North American Chapter of the Association for Computational Linguistics: Human Language Technologies.

[72] Sun X, Ye J, Ren F (2016). Detecting influenza states based on hybrid model with personal emotional factors from social networks. Neurocomputing.

[73] Sun X, Ye J, Ren F (2015). Hybrid model based influenza detection with sentiment analysis from social networks. Proceedings of the 4th National Conference on Social Media Processing.

[74] Yousefinaghani S, Dara R, Poljak Z, Bernardo TM, Sharif S (2019). The assessment of Twitter's potential for outbreak detection: avian influenza case study. Sci Rep.

[75] Rudra K, Sharma A, Ganguly N, Imran M (2018). Classifying and summarizing information from microblogs during epidemics. Inf Syst Front.

[76] Rudra K, Sharma A, Ganguly N, Imran M (2017). Classifying information from microblogs during epidemics. Proceedings of the 2017 International Conference on Digital Health.

[77] Ghosh S, Rudra K, Ghosh S, Ganguly N, Podder S, Balani N, Dubash N (2019). Identifying multi-dimensional information from microblogs during epidemics. Proceedings of the ACM India Joint International Conference on Data Science and Management of Data.

[78] Collier N, Son NT, Nguyen NM (2011). OMG U got flu? Analysis of shared health messages for bio-surveillance. J Biomed Sem.

[79] Sousa L, de Mello R, Cedrim D, Garcia A, Missier P, Uchôa A, Oliveira A, Romanovsky A (2018). VazaDengue: an information system for preventing and combating mosquito-borne diseases with social networks. Inf Syst.

[80] Jain VK, Kumar S (2018). Effective surveillance and predictive mapping of mosquito-borne diseases using social media. J Comput Sci.

[81] Hong Y, Sinnott RO (2018). A social media platform for infectious disease analytics. Proceedings of the 18th International Conference on Computational Science and Its Applications.

[82] Swain S, Seeja KR (2017). Analysis of epidemic outbreak in Delhi using social media data. Proceedings of the Second International Conference on Information, Communication and Computing Technology.

[83] Comito C (2022). Sensing social media to forecast COVID-19 cases. Proceedings of the IEEE Symposium on Computers and Communications.

[84] Perera D, Bamunusinghe J (2022). Contact tracing of covid-19 patients using tweets. Proceedings of the 2nd International Conference on Advanced Research in Computing.

[85] Lazebnik T, Bunimovich-Mendrazitsky S, Ashkenazi S, Levner E, Benis A (2022). Early detection and control of the next epidemic wave using health communications: development of an artificial intelligence-based tool and its validation on COVID-19 data from the US. Int J Environ Res Public Health.

[86] Mundotiya RK, Baruah R, Srivastava B, Singh AK (2020). NLPRL at WNUT-2020 task 2: ELMo-based system for identification of COVID-19 tweets. Proceedings of the Sixth Workshop on Noisy User-Generated Text.

[87] Wang Y, Hao H, Platt LS (2021). Examining risk and crisis communications of government agencies and stakeholders during early-stages of COVID-19 on Twitter. Comput Human Behav.

[88] Cornelius J, Ellendorff T, Furrer L, Rinaldi F (2020). COVID-19 twitter monitor: aggregating and visualizing COVID-19 related trends in social media. Proceedings of the Fifth Social Media Mining for Health Applications Workshop & Shared Task.

[89] Klein AZ, Magge A, O'Connor K, Flores Amaro JI, Weissenbacher D, Gonzalez Hernandez G (2021). Toward using Twitter for tracking COVID-19: a natural language processing pipeline and exploratory data set. J Med Internet Res.

[90] Zhou Y, Jiang JY, Chen X, Wang W (2021). #StayHome or #Marathon?: social media enhanced pandemic surveillance on spatial-temporal dynamic graphs. Proceedings of the 30th ACM International Conference on Information & Knowledge Management.

[91] Lwowski B, Najafirad P (2020). COVID-19 surveillance through Twitter using self-supervised and few shot learning. Proceedings of the 1st Workshop on NLP for COVID-19 (Part 2) at EMNLP 2020.

[92] Amin S, Uddin MI, Zeb MA, Alarood AA, Mahmoud M, Alkinani MH (2020). Detecting dengue/flu infections based on tweets using LSTM and word embedding. IEEE Access.

[93] Khatua A, Khatua A, Cambria E (2019). A tale of two epidemics: contextual Word2Vec for classifying Twitter streams during outbreaks. Inf Process Manage.

[94] Yu X, Xie Z, Mashhadi A, Hong L (2022). Multi-task models for multi-faceted classification of pandemic information on social media. Proceedings of the 14th ACM Web Science Conference 2022.

[95] Wang CK, Singh O, Tang ZL, Dai HJ (2017). Using a recurrent neural network model for classification of tweets conveyed influenza-related information. Proceedings of the International Workshop on Digital Disease Detection using Social Media 2017.

[96] Kia MA, Khaksefidi FE (2022). Twitter flu trend: a hybrid deep neural network for tweet analysis. Proceedings of the 42nd SGAI International Conference on Artificial Intelligence.

[97] Zhao L, Chen J, Chen F, Jin F, Wang W, Lu CT, Ramakrishnan N (2019). Online flu epidemiological deep modeling on disease contact network. Geoinformatica.

[98] Huo HF, Jing SL, Wang XY, Xiang H (2020). Modeling and analysis of a H1N1 model with relapse and effect of Twitter. Physica A.

[99] Huo HF, Zhang XM (2016). Modeling the influence of Twitter in reducing and increasing the spread of influenza epidemics. Springerplus.

[100] Nie Q, Liu Y, Zhang D, Jiang H (2021). Dynamical SEIR model with information entropy using COVID-19 as a case study. IEEE Trans Comput Soc Syst.

[101] Spurlock K, Elgazzar H (2020). Predicting COVID-19 infection groups using social networks and machine learning algorithms. Proceedings of the 11th IEEE Annual Ubiquitous Computing, Electronics & Mobile Communication Conference.

[102] Haupt MR, Jinich-Diamant A, Li J, Nali M, Mackey TK (2021). Characterizing Twitter user topics and communication network dynamics of the “liberate” movement during COVID-19 using unsupervised machine learning and social network analysis. Online Soc Netw Media.

[103] Bagavathi A, Krishnan S, Ahram T (2019). Social sensors early detection of contagious outbreaks in social media. Advances in Artificial Intelligence, Software and Systems Engineering.

[104] Martín G, Marinescu MC, Singh DE, Carretero J (2011). Leveraging social networks for understanding the evolution of epidemics. BMC Syst Biol.

[105] Zheng H, Goh DH, Lee EW, Lee CS, Theng YL (2020). Uncovering topics related to COVID-19 pandemic on Twitter. Proceedings of the 22nd International Conference on Asia-Pacific Digital Libraries.

[106] Shin M, Han S, Park S, Cha M (2020). A risk communication event detection model via contrastive learning. Proceedings of the 3rd NLP4IF Workshop on NLP for Internet Freedom.

[107] Chen L, Hossain KS, Butler P, Ramakrishnan N, Prakash BA (2014). Flu gone viral: syndromic surveillance of flu on Twitter using temporal topic models. Proceedings of the IEEE International Conference on Data Mining.

[108] Fu Q, Hu C, Xu W, He X, Zhang T (2014). Detect and analyze flu outlier events via social network. Proceedings of the Web Technologies and Applications.

[109] Nolasco D, Oliveira J (2019). Subevents detection through topic modeling in social media posts. Future Gener Comput Syst.

[110] Zheng H, Goh DH, Lee CS, Lee EW, Theng YL (2020). Uncovering temporal differences in COVID-19 tweets. Proc Assoc Inf Sci Technol.

[111] Missier P, Romanovsky A, Miu T, Pal A, Daniilakis M, Garcia A, Cedrim D, da Silva Sousa L (2016). Tracking dengue epidemics using Twitter content classification and topic modelling. Proceedings of the Current Trends in Web Engineering.

[112] Odlum M, Yoon S (2015). What can we learn about the Ebola outbreak from tweets?. Am J Infect Control.

[113] Diaz-Aviles E, Stewart A (2012). Tracking Twitter for epidemic intelligence: case study: EHEC/HUS outbreak in Germany, 2011. Proceedings of the 4th Annual ACM Web Science Conference.

[114] Chew C, Eysenbach G (2010). Pandemics in the age of Twitter: content analysis of tweets during the 2009 H1N1 outbreak. PLoS One.

[115] Lehmann BA, Ruiter RA, Kok G (2013). A qualitative study of the coverage of influenza vaccination on Dutch news sites and social media websites. BMC Public Health.

[116] Kobayashi R, Takedomi Y, Nakayama Y, Suda T, Uno T, Hashimoto T, Toyoda M, Yoshinaga N, Kitsuregawa M, Rocha LE (2022). Evolution of public opinion on COVID-19 vaccination in Japan: large-scale Twitter data analysis. J Med Internet Res.

[117] Martin S, Vanderslott S (2022). "Any idea how fast 'It's just a mask!' can turn into 'It's just a vaccine!'": from mask mandates to vaccine mandates during the COVID-19 pandemic. Vaccine.

[118] Zhang C, Xu S, Li Z, Liu G, Dai D, Dong C (2022). The evolution and disparities of online attitudes toward COVID-19 vaccines: year-long longitudinal and cross-sectional study. J Med Internet Res.

[119] Argyris YA, Zhang N, Bashyal B, Tan PN (2022). Using deep learning to identify linguistic features that facilitate or inhibit the propagation of anti- and pro-vaccine content on social media. Proceedings of the IEEE International Conference on Digital Health.

[120] Islam T, Goldwasser D (2022). Understanding COVID-19 vaccine campaign on Facebook using minimal supervision. Proceedings of the IEEE International Conference on Big Data.

[121] Durmaz N, Hengirmen E (2022). The dramatic increase in anti-vaccine discourses during the COVID-19 pandemic: a social network analysis of Twitter. Hum Vaccin Immunother.

[122] Basch CH, Meleo-Erwin Z, Fera J, Jaime C, Basch CE (2021). A global pandemic in the time of viral memes: COVID-19 vaccine misinformation and disinformation on TikTok. Hum Vaccin Immunother.

[123] Kariyapperuma KR, Banujan K, Wijeratna PM, Kumara BT (2022). Classification of covid19 vaccine-related tweets using deep learning. Proceedings of the International Conference on Data Analytics for Business and Industry.

[124] Stella M, Vitevitch MS, Botta F (2022). Cognitive networks extract insights on COVID-19 vaccines from English and Italian popular tweets: anticipation, logistics, conspiracy and loss of trust. Big Data Cogn Comput.

[125] Jabalameli S, Xu Y, Shetty S (2022). Spatial and sentiment analysis of public opinion toward COVID-19 pandemic using twitter data: at the early stage of vaccination. Int J Disaster Risk Reduct.

[126] Feizollah A, Anuar NB, Mehdi R, Firdaus A, Sulaiman A (2022). Understanding COVID-19 halal vaccination discourse on Facebook and Twitter using aspect-based sentiment analysis and text emotion analysis. Int J Environ Res Public Health.

[127] Khan K, Yadav S (2021). Sentiment analysis on covid-19 vaccine using Twitter data: a NLP approach. Proceedings of the IEEE 9th Region 10 Humanitarian Technology Conference.

[128] Ji X, Chun SA, Wei Z, Geller J (2015). Twitter sentiment classification for measuring public health concerns. Soc Netw Anal Min.

[129] Wang S, Schraagen M, Sang ET, Dastani M (2020). Public sentiment on governmental COVID-19 measures in Dutch social media. Proceedings of the 1st Workshop on NLP for COVID-19 (Part 2) at EMNLP 2020.

[130] Zhunis A, Lima G, Song H, Han J, Cha M (2022). Emotion bubbles: emotional composition of online discourse before and after the COVID-19 outbreak. Proceedings of the ACM Web Conference 2022.

[131] Muñoz LM, Ramirez MF, Camargo JE (2020). A data-driven method for measuring the negative impact of sentiment towards China in the context of COVID-19. Proceedings of the Third International Conference on Applied Informatics.

[132] Shah CS, Sebastian MP (2020). Sentiment analysis and topic modelling of Indian government’s Twitter handle #IndiaFightsCorona. Proceedings of the International Conference on Transfer and Diffusion of IT.

[133] Garcia K, Berton L (2021). Topic detection and sentiment analysis in Twitter content related to COVID-19 from Brazil and the USA. Appl Soft Comput.

[134] Massaro M, Tamburro P, La Torre M, Dal Mas F, Thomas R, Cobianchi L, Barach P (2021). Non-pharmaceutical interventions and the infodemic on Twitter: lessons learned from Italy during the covid-19 pandemic. J Med Syst.

[135] Shi J, Li W, Yang Y, Yao N, Bai Q, Yongchareon S, Yu J (2020). Automated concern exploration in pandemic situations - COVID-19 as a use case. Proceedings of the 17th Pacific Rim Knowledge Acquisition Workshop.

[136] Chen L, Huang X, Zhang H, Niu B (2020). Covid-19 public opinion analysis based on LDA topic modeling and data visualization. Proceedings of the Third International Conference on Machine Learning for Cyber Security.

[137] Zhu B, Zheng X, Liu H, Li J, Wang P (2020). Analysis of spatiotemporal characteristics of big data on social media sentiment with COVID-19 epidemic topics. Chaos Solitons Fractals.

[138] Abd-Alrazaq A, Alhuwail D, Househ M, Hamdi M, Shah Z (2020). Top concerns of tweeters during the COVID-19 pandemic: infoveillance study. J Med Internet Res.

[139] Whitfield C, Liu Y, Anwar M (2021). Surveillance of COVID-19 pandemic using social media: a reddit study in North Carolina. Proceedings of the 12th ACM Conference on Bioinformatics, Computational Biology, and Health Informatics.

[140] Kirabo L, Namara M, Mcneese N (2021). The power of the blue tick (): Ugandans’ experiences and engagement on Twitter at the onset of the COVID-19 pandemic. Proceedings of the 3rd African Human-Computer Interaction Conference: Inclusiveness and Empowerment.

[141] Thakur N, Han CY (2022). An exploratory study of tweets about the SARS-CoV-2 Omicron variant: insights from sentiment analysis, language interpretation, source tracking, type classification, and embedded URL detection. COVID.

[142] Avasthi S, Chauhan R, Acharjya DP (2021). Information extraction and sentiment analysis to gain insight into the COVID-19 crisis. Proceedings of the International Conference on Innovative Computing and Communications.

[143] Sabareesha SS, Bhattacharjee S, Shetty RD (2022). Pattern analysis of COVID-19 based on geotagged social media data with sociodemographic factors. Proceedings of the 27th International Conference on Automation and Computing.

[144] Hoque MU, Lee K, Beyer JL, Curran SR, Gonser KS, Lam NS, Mihunov VV, Wang K (2022). Analyzing tweeting patterns and public engagement on Twitter during the recognition period of the COVID-19 pandemic: a study of two U.S. States. IEEE Access.

[145] Koukaras P, Tjortjis C, Rousidis D (2022). Mining association rules from COVID-19 related Twitter data to discover word patterns, topics and inferences. Inf Syst.

[146] Comito C (2022). How COVID-19 information spread in U.S.? The role of Twitter as early indicator of epidemics. IEEE Trans Serv Comput.

[147] Purnat TD, Vacca P, Burzo S, Zecchin T, Wright A, Briand S, Nguyen T (2021). WHO digital intelligence analysis for tracking narratives and information voids in the COVID-19 infodemic. Stud Health Technol Inform.

[148] Lorenzoni V, Andreozzi G, Bazzani A, Casigliani V, Pirri S, Tavoschi L, Turchetti G (2022). How Italy tweeted about COVID-19: detecting reactions to the pandemic from social media. Int J Environ Res Public Health.

[149] Pang PC, Jiang W, Pu G, Chan KS, Lau Y (2022). Social media engagement in two governmental schemes during the COVID-19 pandemic in Macao. Int J Environ Res Public Health.

[150] Abramova O, Batzel K, Modesti D (2022). Collective response to the health crisis among German Twitter users: a structural topic modeling approach. Int J Inf Manage Data Insights.

[151] Wang T, Brooks I, Bashir M (2021). Public reaction on social media during COVID-19: a comparison between Twitter and Weibo. Proceedings of the 2021 Intelligent Computing Conference.

[152] Bogović PK, Meštrović A, Martinčić-Ipšić S (2022). Topic modeling for tracking COVID-19 communication on Twitter. Proceedings of the 28th International Conference on Information and Software Technologies.

[153] León-Sandoval E, Zareei M, Barbosa-Santillán LI, Falcón Morales LE, Pareja Lora A, Ochoa Ruiz G (2022). Monitoring the emotional response to the COVID-19 pandemic using sentiment analysis: a case study in Mexico. Comput Intell Neurosci.

[154] Perera S, Perera I, Ahangama S (2022). Exploring Twitter messages during the COVID-19 pandemic in Sri Lanka: topic modelling and emotion analysis. Proceedings of the 2nd International Conference on Advanced Research in Computing.

[155] Abro HU, Shah ZS, Abbasi H (2022). Analysis of COVID-19 effects on wellbeing - study of Reddit posts using natural language processing techniques. Proceedings of the International Conference on Emerging Trends in Electrical, Control, and Telecommunication Engineering.

[156] Sreeraag G, Shynu PG (2022). A comparative analysis of tweets from the South Indian states based on COVID-19 Omicron wave. Proceedings of the International Conference on Computing, Communication, Security and Intelligent Systems.

[157] Fukuda S, Nanba H, Shoji H (2022). Changes in interests and emotional responses to news coverage of coronavirus disease 2019 case numbers over time. Proceedings of the Joint 12th International Conference on Soft Computing and Intelligent Systems and 23rd International Symposium on Advanced Intelligent Systems.

[158] Nandan M, Mitra S, Dey S (2022). Tweet classification and sentiment analysis of Covid 19 epidemic by applying hybrid based techniques. Proceedings of the IEEE VLSI Device Circuit and System.

[159] Qazi A, Qazi J, Naseer K, Zeeshan M, Khan B, Dey S (2022). Sentiment analysis of nationwide lockdown amid COVID 19: evidence from Pakistan. Proceedings of the IEEE 7th International Conference on Information Technology and Digital Applications.

[160] Pitroda H (2022). Opinion mining approach: to understand public reactions towards reopening of schools and colleges/universities in India. Proceedings of the 2nd International Conference on Innovative Practices in Technology and Management.

[161] Chang A, Xian X, Liu MT, Zhao X (2022). Health communication through positive and solidarity messages amid the COVID-19 pandemic: automated content analysis of Facebook uses. Int J Environ Res Public Health.

[162] Yang W, Wu Z, Mok NY, Ma X (2022). How to save lives with microblogs? Lessons from the usage of Weibo for requests for medical assistance during COVID-19. Proceedings of the 2022 CHI Conference on Human Factors in Computing Systems.

[163] Maylawati DS, Ramdhani MA (2022). Indonesian citizens’ health behavior in a pandemics: Twitter conversation analysis using latent Dirichlet allocation. Proceedings of the 8th International Conference on Wireless and Telematics.

[164] Li L, Aldosery A, Vitiugin F, Nathan N, Novillo-Ortiz D, Castillo C, Kostkova P (2021). The response of governments and public health agencies to COVID-19 pandemics on social media: a multi-country analysis of Twitter discourse. Front Public Health.

[165] Neves JC, de França TC, Bastos MP, de Carvalho PV, Gomes JO (2022). Analysis of government agencies and stakeholders' Twitter communications during the first surge of COVID-19 in Brazil. Work.

[166] Wahbeh A, Nasralah T, Al-Ramahi M, El-Gayar O (2020). Mining physicians' opinions on social media to obtain insights into COVID-19: mixed methods analysis. JMIR Public Health Surveill.

[167] Addawood A, Alsuwailem A, Alohali A, Alajaji D, Alturki M, Alsuhaibani J, Aljabli F (2020). Tracking and understanding public reaction during COVID-19: Saudi Arabia as a use case. Proceedings of the 1st Workshop on NLP for COVID-19 (Part 2) at EMNLP 2020.

[168] Comito C (2021). COVID-19 concerns in US: topic detection in Twitter. Proceedings of the 25th International Database Engineering & Applications Symposium.

[169] Kumari KR, Ratna T, Gayathri T (2022). Machine learning technique with spider monkey optimization for COVID-19 sentiment analysis. Proceedings of the International Conference on Computing, Communication and Power Technology.

[170] Paramarta V, Mailangkay A, Amalia H, Chrismas D (2022). Public sentiment analysis of Indonesian tweets about COVID-19 vaccination using different machine learning approaches. Proceedings of the Seventh International Conference on Informatics and Computing.

[171] Bhatia S, Alhaider M, Alarjani M (2022). Sentiment analysis for Arabic tweets on covid-19 using computational techniques. Proceedings of the 12th International Conference on Cloud Computing, Data Science & Engineering.

[172] Arias F, Guerra-Adames A, Zambrano ME, Quintero-Guerra E, Tejedor-Flores N (2022). Analyzing Spanish-language public sentiment in the context of a pandemic and social unrest: the Panama case. Int J Environ Res Public Health.

[173] Elsaka T, Afyouni I, Hashem I, Al Aghbari Z (2022). Spatio-temporal sentiment mining of COVID-19 Arabic social media. ISPRS Int J Geo Inf.

[174] Rahmanti AR, Chien CH, Nursetyo AA, Husnayain A, Wiratama BS, Fuad A, Yang HC, Li YC (2022). Social media sentiment analysis to monitor the performance of vaccination coverage during the early phase of the national COVID-19 vaccine rollout. Comput Methods Programs Biomed.

[175] Kanakaraddi S, Chikaraddi AK, Aivalli N, Maniyar J, Singh N, Pandian AP, Narayanan M, Palanisamy R, Senjyu T (2022). Sentiment analysis of Covid-19 tweets using machine learning and natural language processing. Proceedings of Third International Conference on Intelligent Computing, Information and Control Systems.

[176] Li L, Zhang Q, Wang X, Zhang J, Wang T, Gao TL, Duan W, Tsoi KK, Wang FY (2020). Characterizing the propagation of situational information in social media during COVID-19 epidemic: a case study on Weibo. IEEE Trans Comput Soc Syst.

[177] Guo R, Xu K (2022). A large-scale analysis of COVID-19 Twitter dataset in a new phase of the pandemic. Proceedings of the IEEE 12th International Conference on Electronics Information and Emergency Communication.

[178] Buvanasri AK, Meenakshi R, Karthika S (2021). Applications of open source intelligence in crisis analysis—a COVID-19 case study. Proceedings of the ICT Systems and Sustainability.

[179] Satu MS, Khan MI, Mahmud M, Uddin S, Summers MA, Quinn JM, Moni MA (2021). TClustVID: a novel machine learning classification model to investigate topics and sentiment in COVID-19 tweets. Knowl Based Syst.

[180] Mandal S, Rath M, Wang Y, Patra BG (2018). Predicting Zika prevention techniques discussed on Twitter: an exploratory study. Proceedings of the 2018 Conference on Human Information Interaction & Retrieval.

[181] Al-Ramahi M, Elnoshokaty A, El-Gayar O, Nasralah T, Wahbeh A (2021). Public discourse against masks in the COVID-19 era: infodemiology study of Twitter data. JMIR Public Health Surveill.

[182] Liang G, Zhao J, Lau HY, Leung CW (2021). Using social media to analyze public concerns and policy responses to COVID-19 in Hong Kong. ACM Trans Manag Inf Syst.

[183] Li L, Hua L, Gao F (2022). Int J Environ Res Public Health.

[184] Mahor K, Manjhvar AK (2022). Public sentiment assessment of coronavirus-specific tweets using a transformer-based BERT classifier. Proceedings of the International Conference on Edge Computing and Applications.

[185] Rifat N, Ahsan M, Gomes R, Chowdhury M (2022). COVID-19 sentiment analysis applying BERT. Proceedings of the IEEE International Conference on Electro Information Technology.

[186] Dewangan A, Sharma A, Badholia A (2022). COVID-19 tweet analysis using deep convolutional neural network (DCNN)-BERT. Proceedings of the 4th International Conference on Inventive Research in Computing Applications.

[187] Stoikos S, Izbicki M (2020). Multilingual emoticon prediction of tweets about COVID-19. Proceedings of the Third Workshop on Computational Modeling of People's Opinions, Personality, and Emotion's in Social Media.

[188] Feldman P, Tiwari S, Cheah CS, Foulds JR, Pan S Analyzing COVID-19 tweets with transformer-based language models. arXiv.

[189] Ghanem A, Asaad C, Hafidi H, Moukafih Y, Guermah B, Sbihi N, Zakroum M, Ghogho M, Dairi M, Cherqaoui M, Baina K (2021). Real-time infoveillance of moroccan social media users' sentiments towards the COVID-19 pandemic and its management. Int J Environ Res Public Health.

[190] Lyu X, Chen Z, Wu D, Wang W (2020). Sentiment analysis on Chinese Weibo regarding COVID-19. Proceedings of the 9th CCF International Conference on Natural Language Processing and Chinese Computing.

[191] Pan Z, Zhou S, Sun N (2022). Twitter sentiment analysis of COVID-19 vaccine based on BiLSTM with attention mechanism. Proceedings of the 4th International Conference on Advances in Computer Technology, Information Science and Communications.

[192] De Santis E, Martino A, Rizzi A (2020). An infoveillance system for detecting and tracking relevant topics from Italian tweets during the COVID-19 event. IEEE Access.

[193] Alsudias L, Rayson P (2020). COVID-19 and Arabic Twitter: how can Arab world governments and public health organizations learn from social media?. Proceedings of the 1st Workshop on NLP for COVID-19 at ACL 2020.

[194] Umer M, Sadiq S, Karamti H, Abdulmajid Eshmawi A, Nappi M, Usman Sana M, Ashraf I (2022). ETCNN: extra tree and convolutional neural network-based ensemble model for COVID-19 tweets sentiment classification. Pattern Recognit Lett.

[195] Malo S, Bayala TR, Kinda Z (2021). French COVID-19 tweets classification using FlauBERT layers. Proceedings of the Congress on Intelligent Systems.

[196] Miao L, Last M, Litvak M (2022). Tracking social media during the COVID-19 pandemic: the case study of lockdown in New York State. Expert Syst Appl.

[197] Luo L, Wang Y, Liu H (2022). COVID-19 personal health mention detection from tweets using dual convolutional neural network. Expert Syst Appl.

[198] Guidry JP, Jin Y, Orr CA, Messner M, Meganck S (2017). Ebola on Instagram and Twitter: how health organizations address the health crisis in their social media engagement. Public Relat Rev.

[199] Roy M, Moreau N, Rousseau C, Mercier A, Wilson A, Atlani-Duault L (2020). Ebola and localized blame on social media: analysis of Twitter and Facebook conversations during the 2014-2015 Ebola epidemic. Cult Med Psychiatry.

[200] Chetty N, Alathur S, Kumar V (2020). 2019-nCoV disease control and rehabilitation: insights from Twitter analytics. Proceedings of the 5th International Conference on Computing, Communication and Security.

[201] Shanthakumar SG, Seetharam A, Ramesh A Understanding the socio-economic disruption in the United States during COVID-19's early days. arXiv.

[202] Niknam F, Samadbeik M, Fatehi F, Shirdel M, Rezazadeh M, Bastani P (2021). COVID-19 on Instagram: a content analysis of selected accounts. Health Policy Technol.

[203] Essam BA, Abdo MS (2021). How do Arab tweeters perceive the COVID-19 pandemic?. J Psycholinguist Res.

[204] Ölcer S, Yilmaz-Aslan Y, Brzoska P (2020). Lay perspectives on social distancing and other official recommendations and regulations in the time of COVID-19: a qualitative study of social media posts. BMC Public Health.

[205] Yigitcanlar T, Kankanamge N, Preston A, Gill PS, Rezayee M, Ostadnia M, Xia B, Ioppolo G (2020). How can social media analytics assist authorities in pandemic-related policy decisions? Insights from Australian states and territories. Health Inf Sci Syst.

[206] Xia H, An W, Li J, Zhang ZJ (2022). Outlier knowledge management for extreme public health events: understanding public opinions about COVID-19 based on microblog data. Socioecon Plann Sci.

[207] Huang F, Ding H, Liu Z, Wu P, Zhu M, Li A, Zhu T (2020). How fear and collectivism influence public's preventive intention towards COVID-19 infection: a study based on big data from the social media. BMC Public Health.

[208] Machete P, Turpin M (2020). The use of critical thinking to identify fake news: a systematic literature review. Responsible Design, Implementation and Use of Information and Communication Technology.

[209] Guo Q, Huang W, Huang K, Liu X (2015). Information credibility: a probabilistic graphical model for identifying credible influenza posts on social media. Proceedings of the International Conference on Smart Health.

[210] Abdelminaam D, Ismail F, Taha M, Taha A, Houssein E, Nabil A (2021). CoAID-DEEP: an optimized intelligent framework for automated detecting COVID-19 misleading information on Twitter. IEEE Access.

[211] Madani Y, Erritali M, Bouikhalene B (2021). Using artificial intelligence techniques for detecting Covid-19 epidemic fake news in Moroccan tweets. Results Phys.

[212] Cui L, Lee D Coaid: Covid-19 healthcare misinformation dataset. arXiv.

[213] Hossain T, Logan RL IV, Ugarte A, Matsubara Y, Young S, Singh S (2020). COVIDLies: detecting COVID-19 misinformation on social media. Proceedings of the 1st Workshop on NLP for COVID-19 (Part 2) at EMNLP 2020.

[214] Malla S, Alphonse PJ (2022). Fake or real news about COVID-19? Pretrained transformer model to detect potential misleading news. Eur Phys J Spec Top.

[215] Alajramy L, Jarrar R (2022). Using artificial neural networks to identify COVID-19 misinformation. Proceedings of the 4th Multidisciplinary International Symposium on Disinformation in Open Online Media.

[216] Samantaray S, Kumar A (2021). Bi-directional long short-term memory network for fake news detection from social media. Proceedings of the Intelligent and Cloud Computing.

[217] Ghayoomi M, Mousavian M (2022). Deep transfer learning for COVID-19 fake news detection in Persian. Expert Syst.

[218] Raj C, Meel P (2022). ARCNN framework for multimodal infodemic detection. Neural Netw.

[219] Tang C, Ma K, Cui B, Ji K, Abraham A (2022). Long text feature extraction network with data augmentation. Appl Intell (Dordr).

[220] Zuo C, Banerjee R, Chaleshtori FH, Shirazi H, Ray I (2022). Seeing should probably not be believing: the role of deceptive support in COVID-19 misinformation on Twitter. J Data Inf Quality.

[221] Biradar S, Saumya S, Chauhan A (2023). Combating the infodemic: COVID-19 induced fake news recognition in social media networks. Complex Intell Systems.

[222] Jeyasudha J, Seth P, Usha G, Tanna P (2022). Fake information analysis and detection on pandemic in Twitter. SN Comput Sci.

[223] Alouffi B, Alharbi A, Sahal R, Saleh H (2021). An optimized hybrid deep learning model to detect COVID-19 misleading information. Comput Intell Neurosci.

[224] Hussna A, Trisha II, Karim MS, Alam MG (2021). COVID-19 fake news prediction on social media data. Proceedings of the IEEE Region 10 Symposium.

[225] Ajao O, Garg A, Da Costa-Abreu M (2022). Exploring content-based and meta-data analysis for detecting fake news infodemic: a case study on COVID-19. Proceedings of the 12th International Conference on Pattern Recognition Systems.

[226] Alhakami H, Alhakami W, Baz A, Faizan M, Khan MW, Agrawal A (2022). Evaluating intelligent methods for detecting COVID-19 fake news on social media platforms. Electronics.

[227] Ali M, Murtza I, Ejaz A (2021). Pandemic rumor identification on social networking sites: a case study of COVID-19. Proceedings of the 2021 5th International Conference on Natural Language Processing and Information Retrieval.

[228] Maakoul O, Boucht S, Hachimi KE, Azzouzi S (2020). Towards evaluating the COVID’19 related fake news problem: case of Morocco. Proceedings of the IEEE 2nd International Conference on Electronics, Control, Optimization and Computer Science.

[229] Mehta V, Mishra RK (2021). Machine learning based fake news detection on Covid-19 tweets data. Proceedings of International Conference on Computational Intelligence and Data Engineering.

[230] Khan S, Hakak S, Deepa N, Prabadevi B, Dev K, Trelova S (2021). Detecting COVID-19-related fake news using feature extraction. Front Public Health.

[231] Jouyandeh F, Rahmatikargar S, Rahmatikargar B, Zadeh PM (2021). Fake news and COVID-19 vaccination: a comparative study. Proceedings of the 2021 IEEE/ACM International Conference on Advances in Social Networks Analysis and Mining.

[232] Bamiro B, Assayad I (2021). Data-based automatic Covid-19 rumors detection in social networks. Proceedings of the Networking, Intelligent Systems and Security.

[233] Zhang Y, Guo B, Ding Y, Liu J, Qiu C, Liu S, Yu Z (2022). Investigation of the determinants for misinformation correction effectiveness on social media during COVID-19 pandemic. Inf Process Manag.

[234] Belay EG, Beyene M, Alemu T, Negash A, Tesema T, Mohammed A, Yilma M, Tassew B, Mekonnen S (2020). Towards curtailing infodemic in the era of COVID-19: a contextualized solution for Ethiopia. Proceedings of the HCI International 2020 – Late Breaking Papers: Interaction, Knowledge and Social Media.

[235] Sell TK, Hosangadi D, Trotochaud M (2020). Misinformation and the US Ebola communication crisis: analyzing the veracity and content of social media messages related to a fear-inducing infectious disease outbreak. BMC Public Health.

[236] Alsudias L, Rayson P (2019). Classifying information sources in Arabic Twitter to support online monitoring of infectious diseases. Proceedings of the 3rd Workshop on Arabic Corpus Linguistics.

[237] Jain L (2022). An entropy-based method to control COVID-19 rumors in online social networks using opinion leaders. Technol Soc.

[238] Chen B, Chen X, Pan J, Liu K, Xie B, Wang W, Peng Y, Wang F, Li N, Jiang J (2021). Dissemination and refutation of rumors during the COVID-19 outbreak in China: infodemiology study. J Med Internet Res.

[239] Zeng J, Zhang Y, Ma X (2021). Fake news detection for epidemic emergencies via deep correlations between text and images. Sustain Cities Soc.

[240] Atehortua NA, Patino S (2021). COVID-19, a tale of two pandemics: novel coronavirus and fake news messaging. Health Promot Int.

[241] Madraki G, Grasso I, Otala JM, Liu Y, Matthews J (2021). Characterizing and comparing COVID-19 misinformation across languages, countries and platforms. Proceedings of the Companion Proceedings of the Web Conference 2021.

[242] Raj C, Meel P (2022). People lie, actions Don't! Modeling infodemic proliferation predictors among social media users. Technol Soc.

[243] Heidari M, Zad S, Hajibabaee P, Malekzadeh M, HekmatiAthar S, Uzuner O (2021). BERT model for fake news detection based on social bot activities in the COVID-19 pandemic. Proceedings of the IEEE 12th Annual Ubiquitous Computing, Electronics & Mobile Communication Conference.

[244] Yan M, Lin YR, Chung WT (2022). Are mutated misinformation more contagious? A case study of COVID-19 misinformation on Twitter. Proceedings of the 14th ACM Web Science Conference 2022.

[245] Shahi GK, Dirkson A, Majchrzak TA (2021). An exploratory study of COVID-19 misinformation on Twitter. Online Soc Netw Media.

[246] Valecha R, Srinivasan SK, Volety T, Kwon KH, Agrawal M, Rao HR (2021). Fake news sharing: an investigation of threat and coping cues in the context of the Zika virus. Digital Threats.

[247] Singh L, Bode L, Budak C, Kawintiranon K, Padden C, Vraga E (2020). Understanding high- and low-quality URL sharing on COVID-19 Twitter streams. J Comput Soc Sci.

[248] Sacco PL, Gallotti R, Pilati F, Castaldo N, De Domenico M (2021). Emergence of knowledge communities and information centralization during the COVID-19 pandemic. Soc Sci Med.

[249] Ermakova L, Nurbakova D, Ovchinnikova I (2020). Covid or not Covid? Topic shift in information cascades on Twitter. Proceedings of the 3rd International Workshop on Rumours and Deception in Social Media.

[250] Fan Y (2020). The social amplification of risk on Weibo-take the COVID-19 epidemic in China as an example. Proceedings of the 2020 Conference on Artificial Intelligence and Healthcare.

[251] Chen E, Lerman K, Ferrara E (2020). Tracking social media discourse about the COVID-19 pandemic: development of a public coronavirus Twitter data set. JMIR Public Health Surveill.

[252] Bahja M, Safdar GA (2020). Unlink the link between COVID-19 and 5G networks: an NLP and SNA based approach. IEEE Access.

[253] Ahmed W, Vidal-Alaball J, Downing J, López Seguí F (2020). COVID-19 and the 5G conspiracy theory: social network analysis of Twitter data. J Med Internet Res.

[254] Shahsavari S, Holur P, Wang T, Tangherlini TR, Roychowdhury V (2020). Conspiracy in the time of corona: automatic detection of emerging COVID-19 conspiracy theories in social media and the news. J Comput Soc Sci.

[255] Lanier HD, Diaz MI, Saleh SN, Lehmann CU, Medford RJ (2022). Analyzing COVID-19 disinformation on Twitter using the hashtags #scamdemic and #plandemic: retrospective study. PLoS One.

[256] Ahmed W, López Seguí F, Vidal-Alaball J, Katz MS (2020). COVID-19 and the "film your hospital" conspiracy theory: social network analysis of Twitter data. J Med Internet Res.

[257] Li S, Wang Y, Xue J, Zhao N, Zhu T (2020). The impact of COVID-19 epidemic declaration on psychological consequences: a study on active Weibo users. Int J Environ Res Public Health.

[258] Fatimah N, Budi I, Santoso AB, Putra PK (2021). Analysis of mental health during the Covid-19 pandemic in Indonesia using Twitter data. Proceedings of the 8th International Conference on Advanced Informatics: Concepts, Theory and Applications.

[259] Davis BD, McKnight DE, Teodorescu D, Quan-Haase A, Chunara R, Fyshe A, Lizotte DJ (2020). Quantifying depression-related language on social media during the COVID-19 pandemic. Int J Popul Data Sci.

[260] Wolohan JT (2020). Estimating the effect of COVID-19 on mental health: linguistic indicators of depression during a global pandemic. Proceedings of the 1st Workshop on NLP for COVID-19 at ACL 2020.

[261] Chen JQ, Qi K, Zhang A, Shalaginov M, Zeng TH (2022). COVID-19 impact on mental health analysis based on Reddit comments. Proceedings of the IEEE International Conference on Bioinformatics and Biomedicine.

[262] Nandy S, Kumar V (2021). My mind is a prison: a boosted deep learning approach to detect the rise in depression since COVID-19 using a stacked bi-LSTM CatBoost model. Proceedings IEEE International Conference on Big Data.

[263] Viviani M, Crocamo C, Mazzola M, Bartoli F, Carrà G, Pasi G (2021). Assessing vulnerability to psychological distress during the COVID-19 pandemic through the analysis of microblogging content. Future Gener Comput Syst.

[264] Kumar R, Mukherjee S, Choi TM, Dhamotharan L (2022). Mining voices from self-expressed messages on social-media: diagnostics of mental distress during COVID-19. Decis Support Syst.

[265] Gao J, Zheng P, Jia Y, Chen H, Mao Y, Chen S, Wang Y, Fu H, Dai J (2020). Mental health problems and social media exposure during COVID-19 outbreak. PLoS One.

[266] Pang H (2021). How compulsive WeChat use and information overload affect social media fatigue and well-being during the COVID-19 pandemic? A stressor-strain-outcome perspective. Telemat Inform.

[267] Ni MY, Yang L, Leung CM, Li N, Yao XI, Wang Y, Leung GM, Cowling BJ, Liao Q (2020). Mental health, risk factors, and social media use during the COVID-19 epidemic and cordon sanitaire among the community and health professionals in Wuhan, China: cross-sectional survey. JMIR Ment Health.

[268] Neill R, Blair C, Best P, McGlinchey E, Armour C Media consumption and mental health during COVID-19 lockdown: a UK cross-sectional study across England, Wales, Scotland and Northern. PsyArXiv Preprints.

[269] Chao M, Xue D, Liu T, Yang H, Hall BJ (2020). Media use and acute psychological outcomes during COVID-19 outbreak in China. J Anxiety Disord.

[270] Daimer S, Mihatsch LL, Neufeld SA, Murray GK, Knolle F (2022). Investigating the relationship of COVID-19 related stress and media consumption with schizotypy, depression, and anxiety in cross-sectional surveys repeated throughout the pandemic in Germany and the UK. Elife.

[271] Yu L, Du M (2022). Social networking use, mental health, and quality of life of Hong Kong adolescents during the COVID-19 pandemic. Front Public Health.

[272] Brailovskaia J, Margraf J (2022). Positive mental health and mindfulness as protective factors against addictive social media use during the COVID-19 outbreak. PLoS One.

[273] Zhao N, Zhou G (2020). Social media use and mental health during the COVID-19 pandemic: moderator role of disaster stressor and mediator role of negative affect. Appl Psychol Health Well Being.

[274] Lazer D, Kennedy R, King G, Vespignani A (2014). Big data. The parable of Google Flu: traps in big data analysis. Science.

[275] ChaiAsaad / SLR_EpidemicsAndSocialMedia. GitHub.

